# Differential Domain Distribution of gnomAD- and Disease-Linked Connexin Missense Variants

**DOI:** 10.3390/ijms22157832

**Published:** 2021-07-22

**Authors:** Donglin Bai, Jiayi Wang, Tianhe Li, Ryan Chan, Mena Atalla, Robert C. Chen, Mohammad T. Khazaneh, Raphael J. An, Peter B. Stathopulos

**Affiliations:** Department of Physiology and Pharmacology, University of Western Ontario, London, ON N6A 5C1, Canada; jwan625@uwo.ca (J.W.); tli643@uwo.ca (T.L.); rchan282@uwo.ca (R.C.); matalla6@uwo.ca (M.A.); rche5152@uni.sydney.edu.au (R.C.C.); mtahvil@uwo.ca (M.T.K.); jan54@uwo.ca (R.J.A.); Peter.Stathopulos@schulich.uwo.ca (P.B.S.)

**Keywords:** gap junction structure, connexin variants, Cx43, Cx40, Cx46, Cx50, evolutionary co-variation analysis, congenital cataracts, atrial fibrillation, oculodentodigital dysplasia

## Abstract

Twenty-one human genes encode connexins, a family of homologous proteins making gap junction (GJ) channels, which mediate direct intercellular communication to synchronize tissue/organ activities. Genetic variants in more than half of the connexin genes are associated with dozens of different Mendelian inherited diseases. With rapid advances in DNA sequencing technology, more variants are being identified not only in families and individuals with diseases but also in people in the general population without any apparent linkage to Mendelian inherited diseases. Nevertheless, it remains challenging to classify the pathogenicity of a newly identified connexin variant. Here, we analyzed the disease- and Genome Aggregation Database (gnomAD, as a proxy of the general population)-linked variants in the coding region of the four disease-linked α connexin genes. We found that the most abundant and position-sensitive missense variants showed distinct domain distribution preference between disease- and gnomAD-linked variants. Plotting missense variants on topological and structural models revealed that disease-linked missense variants are highly enriched on the structurally stable/resolved domains, especially the pore-lining domains, while the gnomAD-linked missense variants are highly enriched in the structurally unstable/unresolved domains, especially the carboxyl terminus. In addition, disease-linked variants tend to be on highly conserved residues and those positions show evolutionary co-variation, while the gnomAD-linked missense variants are likely on less conserved residue positions and on positions without co-variation. Collectively, the revealed distribution patterns of disease- and gnomAD-linked missense variants further our understanding of the GJ structure–biological function relationship, which is valuable for classifying the pathogenicity of newly identified connexin variants.

## 1. Introduction

Gap junction (GJ) channels mediate direct intercellular communication to synchronize tissue/organ activities electrically and metabolically. A GJ channel is formed by proper docking of two hemichannels (also known as connexons), and each hemichannel is a hexamer of connexin molecules. Twenty-one genes in the human genome encode connexin molecules, which could be further classified into five groups according to their sequence similarities: α (seven connexins), β (seven connexins), γ (three connexins), δ (three connexins), and ε (one connexin). Every connexin shows a unique tissue distribution pattern, and every cell often expresses more than one type of connexin [1,2]. GJs in cells from the same or different tissues could be homomeric homotypic, homomeric heterotypic, and/or heteromeric heterotypic GJs. Functionally, GJs mediate rapid electrical signals and/or allow the permeation of nutrients, signaling molecules, and metabolic wastes with molecular weights of less than 1 kilodalton [1].

Previous structural studies suggested that the gap junction channel is formed by two head-to-head docked hexameric connexons, and the transmembrane domains are formed by α helical structures [3,4,5]. The precise three-dimensional structure of specific connexin domains such as the critical pore-forming region in relation to the membrane was not clear until high-resolution structures became available. The first high-resolution (3.4 Å) GJ structure, which was resolved more than ten years ago, was of the human β connexin, Cx26 [6,7]. Recently, the GJ structures of α connexins (sheep Cx46 and Cx50) were resolved using cryogenic electron microscopy (cryo-EM) [8]. These high-resolution structure models made studies on the structure–biological function relationship possible on these and other closely related α and β connexins.

Genetic variations in more than half of human connexin genes have been linked to Mendelian inherited diseases [9,10]. Depending on the connexin and the linked disease, the inheritance could be autosomal dominant or autosomal recessive as well as X-linked. Rapid advances of DNA sequencing technology greatly accelerated the identification of genetic variations underlying Mendelian inherited diseases, including many in the connexin genes. A much greater number of disease-linked variants have now been identified in connexin genes, and analyzing their domain distribution is now possible as an important disease prognosticator. Parallel to this development, several large-scale studies focused their efforts on sequencing human exomes and/or genomes from a large number of individuals of different ethnic origin, without any apparent link to Mendelian diseases [11,12,13]. The Genome Aggregation Database (gnomAD) collected high-quality sequences of over 125,748 exomes and 15,708 genomes from several different populations [13,14]. Within this database, over 17 million variants have been identified in the exomes, and many of them are in the coding regions, which could alter the amino acid type, structure, and function of the protein.

In addition to connexins from the human genome, numerous connexin genes from other vertebrate species have been sequenced [15,16,17]. It has been known for decades that the alignment of connexin sequences reveals highly conserved domains (e.g., transmembrane domains and extracellular loops) and less conserved domains (e.g., intracellular loop and carboxyl terminus) as well as connexin group-specific sequence signatures [15,16,18]. The existence of highly conserved connexin residues argues for their important biological role in connexin structure and function, and mutations of these residues are likely to lead to diseases. Based on such a dichotomy, the evolution conservation scores for protein residues have been developed and widely used as predictors for assessing potential deleterious effects [19]. Interestingly, some protein residue variations are frequently coupled with a network of residue changes [20]. This co-variation also likely reveals residues essential for connexin structure and function [20]. Yet, it remains uncertain if connexin disease-linked variants show preferential localization on highly conserved residues and those residues show evolutionary co-variation. Furthermore, the localization pattern of connexin variants listed in the reference genome databases remain relatively unexamined [11,12,13,14].

We surveyed the coding region variants in the four α connexins that have been linked with inherited diseases and found that the number of unique variants in gnomAD has already surpassed the known disease-linked variants. However, despite the availability of this information, it is not clear if there are any general protein domain distribution preferences between the disease-linked variants compared to the apparently non-disease-linked (i.e., gnomAD-linked) variants. Therefore, here we collated 218 disease-linked and 1439 gnomAD-linked connexin variants of four α connexin genes and analyzed the variant type and the domain distribution. Comparing the disease-linked with gnomAD-linked variants, we revealed a remarkably segregated distribution of these variants on available structure models. Collectively, our work provides an initial basis for understanding the importance of different connexin domains in terms of tolerance to genetic variations and will be a useful tool for pathogenicity classification of newly identified disease-linked variants.

## 2. Results

### 2.1. Gap Junction Structures and Conservation of Human α Connexins

**Figure 1:**
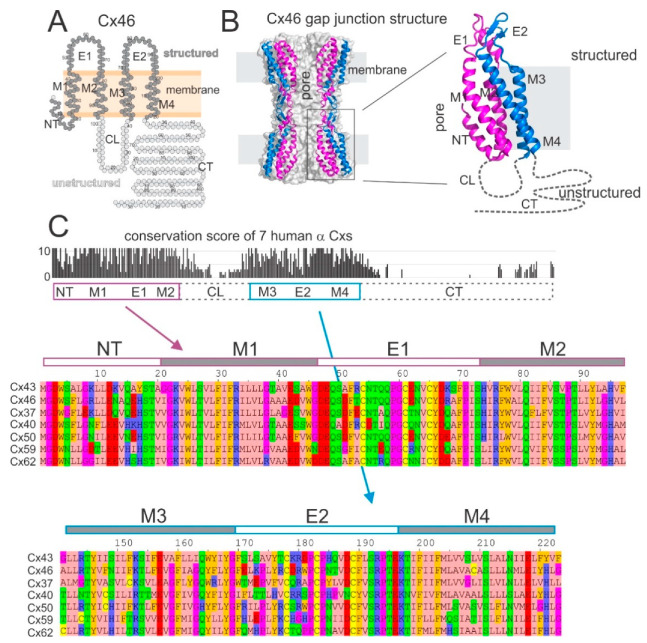
Gap junction structure and sequence alignment in structure resolved domains in α-connexins. (**A**) Topological structure of sheep Cx46 showing four transmembrane domains (M1–4), two extracellular loops (E1 and E2), one cytoplasmic loop (CL), amino terminus (NT), and carboxyl terminus (CT). The structured residues are colored in dark gray, and the unstructured residues are colored in light gray. (**B**) Sheep Cx46 gap junction structure (6MHQ) is shown in a cartoon representation with PyMOL [21]. Four connexin subunits out of 12 are shown (two on each side) to highlight the pore. Pore-lining domains (NT, M1, E1, and M2 or called pore domain) are colored in magenta and the domains on the periphery of the channel (M3, E2, and M4, called non-pore domain) are colored in blue. An enlarged view of a single Cx46 subunit is shown on the right to display the domains in structure-resolved domains and the structure-unresolved domains (CL and CT by dashed lines). (**C**) Structured domains are highly conserved among seven human α-connexins. Seven human α-connexins are aligned and they displayed high conservation in the structured domains on both the pore (magenta-colored box, including NT, M1, E1, and M2) and non-pore (blue-colored box, including M3, E2, and M4) domains using the conservation scores of Jalview [22]. Note only the beginning portion of the CT domain conservation is shown.

All connexins, including human α connexins, are predicted to have the same topological structure with four transmembrane domains (M1–M4), two extracellular loops (E1, E2), one cytoplasmic loop (CL), and both amino terminus (NT) and carboxyl terminus (CT) on the cytosol side of plasma membrane. Topological and three-dimensional GJ structures of sheep Cx46 are illustrated in Figure 1A,B, respectively, to highlight the relative positions of the domains. The structure-resolved (or structured) and presumably most stable domains in each connexin subunit, as shown in Figure 1B, includes two pieces. The first piece, containing NT, M1, E1, and M2 domains, directly faces the GJ pore (defined as pore domains, colored in magenta in Figure 1B). The second piece, containing M3, E2, and M4 domains, faces away from the GJ pore (defined as non-pore domains, colored in blue in Figure 1B). Both the CL and CT domains are not structurally resolved (or unstructured) and are presumed to be unstable and/or disordered (shown as either light gray circles in Figure 1A or dashed lines in Figure 1B).

Protein sequence alignment of all seven human α connexins (Figure 1C) revealed that the two pieces corresponding to those structured domains are highly conserved, while the CL and CT domains displayed a much lower level of conservation (Figure 1C top). Many amino acid residues are either identical or have similar physicochemical properties in the two structure-resolved pieces of these α connexins (Figure 1C bottom), suggesting that all these human α connexins likely have a similar structure as that of the Cx46. Additionally, human Cx43, Cx46, Cx40, and Cx50 showed high protein sequence identity (42%, 67%, 45%, and 47%, respectively overall as well as 67%, 96%, 69%, and 80%, respectively within the structured domains) with sheep Cx46, arguing that the sheep Cx46 structure model is an excellent template for these human α connexins.

### 2.2. Variants Linked to Diseases vs. Variants Found in gnomAD

Genetic variants in four α connexin genes have been linked to inherited diseases. We compiled a total of 218 variants from 150 articles (Tables S1–S4). Among them, eighty-nine variants in *GJA1* (Table S1) were found to be linked to oculodentodigital dysplasia (ODDD). The *GJA1* gene encodes Cx43, which is the most widely expressed connexin in our body. One hundred sixteen congenital cataract-linked variants in *GJA3* (Table S2) and *GJA8* are known (Table S3), which are the two genes encoding the lens connexins, Cx46 and Cx50, respectively. Early onset atrial fibrillation (AF) linked variants (Table S4) were found in *GJA5* (13 currently known), which encode a connexin, Cx40, abundantly expressed in several tissues including the atria of the heart. Most of these disease-linked variants are inherited autosomal dominantly with the pattern of inheritance for some sporadic cases not yet fully clear. Autosomal recessive cases in ODDD and cataracts were also reported in rare occasions (see Tables S1–S3).

**Figure 2:**
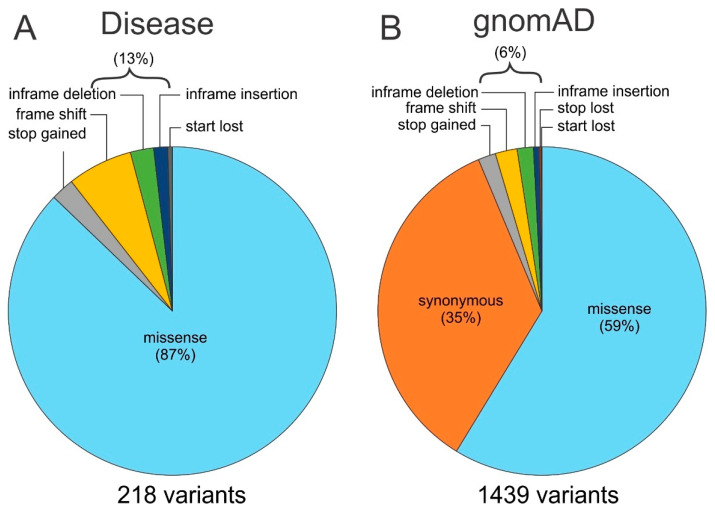
Pie charts to show proportions of different types of variants. The total number, type, and their relative percentage (calculated by the number of unique variants for each variant type) in four α-connexins (Cx43, Cx46, Cx40, and Cx50) are shown as pie charts. (**A**) The pie chart illustrates the disease-linked variants. (**B**) The pie chart shows the variants identified in the reference genome database, gnomAD (V2.1.1). Synonymous variants were not reported from the disease-linked variants, and in both cases, the number of unique missense variants showed the highest percentage.

Sequencing of control individuals with no apparent association with any known Mendelian inherited diseases was conducted, and the discovered variants are readily available in publicly accessible databases, including those identified in the Exome Sequencing Project, the 1000 genome project, the Exome Aggregation Consortium (ExAC), and the Genome Aggregation Database (gnomAD) [11,12,13,14]. Side to side pie charts were created to summarize the proportion of different types of unique variants found in the disease-linked cohort and those found in gnomAD for the four α connexins (Figure 2).

**Figure 3:**
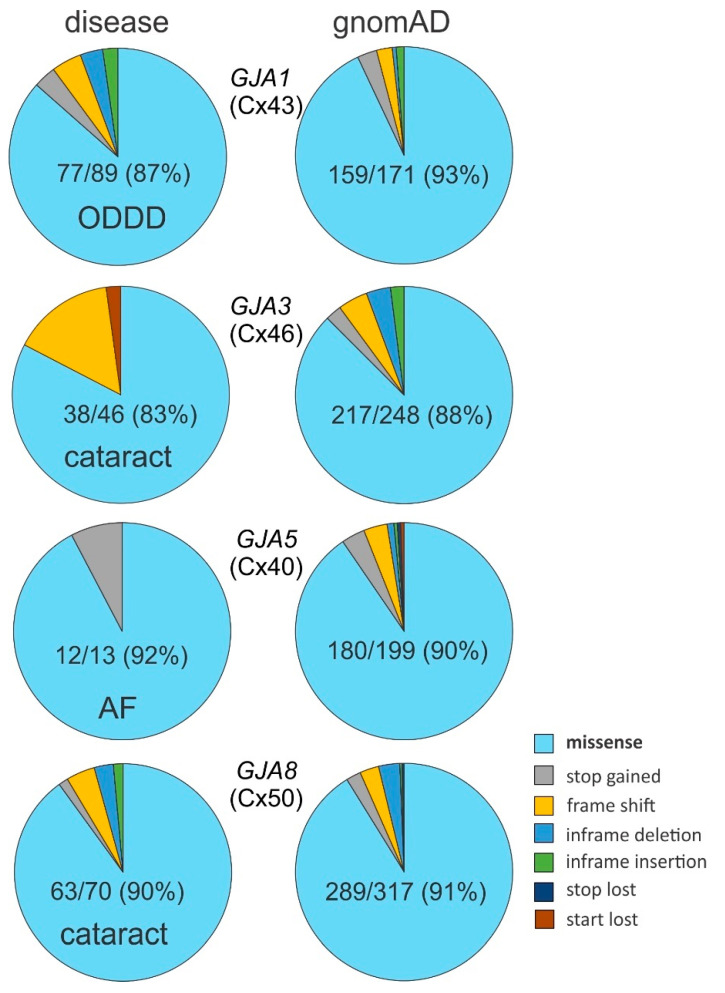
Missense variant is dominant among all variant types. Pie charts to illustrate that the number of unique missense variants is dominant in all variants observed in these four connexins irrespective to disease- or gnomAD-linked. Disease-linked variants pie charts are on the left (disease name is indicated on each pie) and the gnomAD-linked variants pie charts are on the right. Missense variants (light blue color) and their percentages are indicated. The relative distribution of disease-linked missense variants is not statistically different from that of gnomAD-linked missense variants for each of these connexins (Fisher’s exact test, *p* > 0.05 for each pair). Note, the synonymous variants were removed from the gnomAD-linked variants for easier comparison with those disease-linked variants. AF, atrial fibrillation; ODDD, oculodentodigital dysplasia.

Most of the disease-linked variants are missense variants (87%) involving only an exchange of a single amino acid residue. The remaining variant types are stop gained (also known as nonsense), frame shift, in-frame deletion or insertion (these two also known as indels), and start lost (Figure 2 and Table S5). In contrast, the same four genes encoding Cx43, Cx46, Cx50, and Cx40 show many more genetic variants (a total of 1439) within the coding regions in gnomAD, with 59% being missense variants. Synonymous variants account for about one-third of the variants (35%), which was not reported among disease-linked variants, likely because the amino acid residues remain unchanged. The remaining variant types (6%) are very much the same as those reported for disease-linked with one addition, stop lost (Figure 2 and Table S5). We removed the synonymous variants from the gnomAD-linked variants for easier direct comparisons between the disease- and gnomAD-linked variant type as well as relative percentages of the unique variant numbers in each of these connexins. We found that the total number of unique variants was wide-ranging, from 13 AF-linked Cx40 variants to 317 variants found in gnomAD-linked Cx50 variants. Nevertheless, some common patterns were revealed: the percentages of missense variants are consistently high and dominating in every pie chart (from 83% to 93%, Figure 3) and conversely, the other variants (stop gained, frame shift, indels, stop lost, start lost) only exist in a small fraction (7–17%) of the pie chart with one or more types completely missing probably due to the rareness in the limited number of cases (Figure 3).

We focused our subsequent efforts on examining the missense variants for several reasons. First, missense variants are the most prevalent variant type in both disease-linked and gnomAD-linked variants, as shown in Figure 3. In most of these α connexins, the total number of unique missense variants have already surpassed several dozen in both the disease- and gnomAD-linked cohort, with the exception of AF-linked Cx40 variants. With this level of prevalence, we have a compelling sample representation to analyze domain preference. Second, missense variants carry important positional information for the residue or domain without changing the overall protein length. These properties enable accurate mapping of the variants on topological and structural models (see below). Enrichment of disease-linked missense variants in a connexin domain indicates the essentiality of the domain in normal function. On the other hand, depletion or reduction of gnomAD-linked missense variants in a connexin domain, which is a reflection of the general population, would also indicate that this domain is functionally important and less tolerant to missense variations. The reverse, or depletion of disease-linked and enrichment of gnomAD-linked missense variant in a connexin domain, would suggest that this domain may be less critical for normal biology, therefore containing more prevalent changes in the general population. Finally, other variant types involve either increases or decreases in protein length or shifting multiple residues in the protein domains, making these types of mutants difficult to analyze with respect to topological or structure models.

### 2.3. Domain Localizations of Disease- and gnomAD-Linked Missense Variants

**Figure 4:**
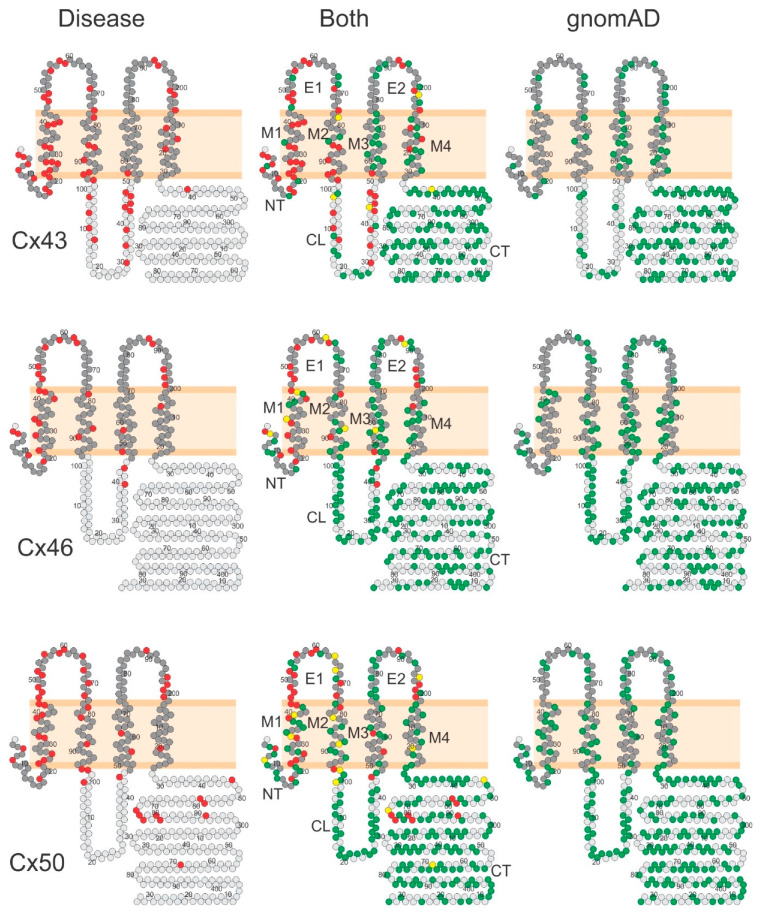
Disease-linked and gnomAD-linked missense variants on topological structure models. Topological structure models for human Cx43, Cx46, and Cx50 are shown. The residues corresponding to structure-resolved (dark gray) or structure-unresolved residues (light gray) are indicated. Left panels illustrate the disease-linked missense variant residues in red, and the right panels display the gnomAD-linked missense variant residues in green. The middle panels illustrate both disease-linked missense variants and gnomAD-linked missense variants (the overlapped residues are colored in yellow). Among these overlapped positions, most of the disease-linked missense variants (22/26) were different amino acid substitutions from those found in gnomAD. The differential distribution patterns are discussed below. Note some residue positions can have two or more unique missense variants.

To visualize the domain locations of missense variants, we first aligned the connexin protein sequences of human Cx43, Cx46, and Cx50 with sheep Cx46 and then plotted missense variants onto topological structure models as shown in Figure 4. The residues in the equivalent structured domains (NT, M1, E1, M2, M3, E2, M4) are represented by dark gray circles, and the residues in structure unresolved (unstructured) domains (CL and CT) are shown as light gray circles (Figure 4). Then, we colored the disease-linked missense variant residue positions in red (Figure 4 left panels), the gnomAD-linked missense variant residue positions in green (Figure 4 right panels), and those positions with both types of variants in yellow (Figure 4 middle panels; among the 26 overlapped positions, 18 are in the structured domains, and 8 are in the unstructured domains). A total of 26 residue positions showed 35 disease-linked and 31 gnomAD-linked variants (see Figure 4, middle panel, yellow-colored positions). It is interesting to note that the majority (28/35) of the disease-linked missense variants on these 26 overlapped residue positions are different amino acid substitutions from those in gnomAD-linked missense variants. Only a minority of the missense variants (7/35) were identical residue substitutions for both the disease- and gnomAD-linked variants. Due to the low number (12) of AF-linked Cx40 missense variants, the distribution of Cx40 missense variants was not included in this figure.

A couple of notable patterns of distribution were apparent for these missense variants. First, the disease-linked variants are much more frequently on residues in the structured domains (dark gray circles in Figure 4). Second, the gnomAD-linked variants are much more likely on the residues in the structurally unresolved or unstructured domains (light gray circles in Figure 4).

**Figure 5:**
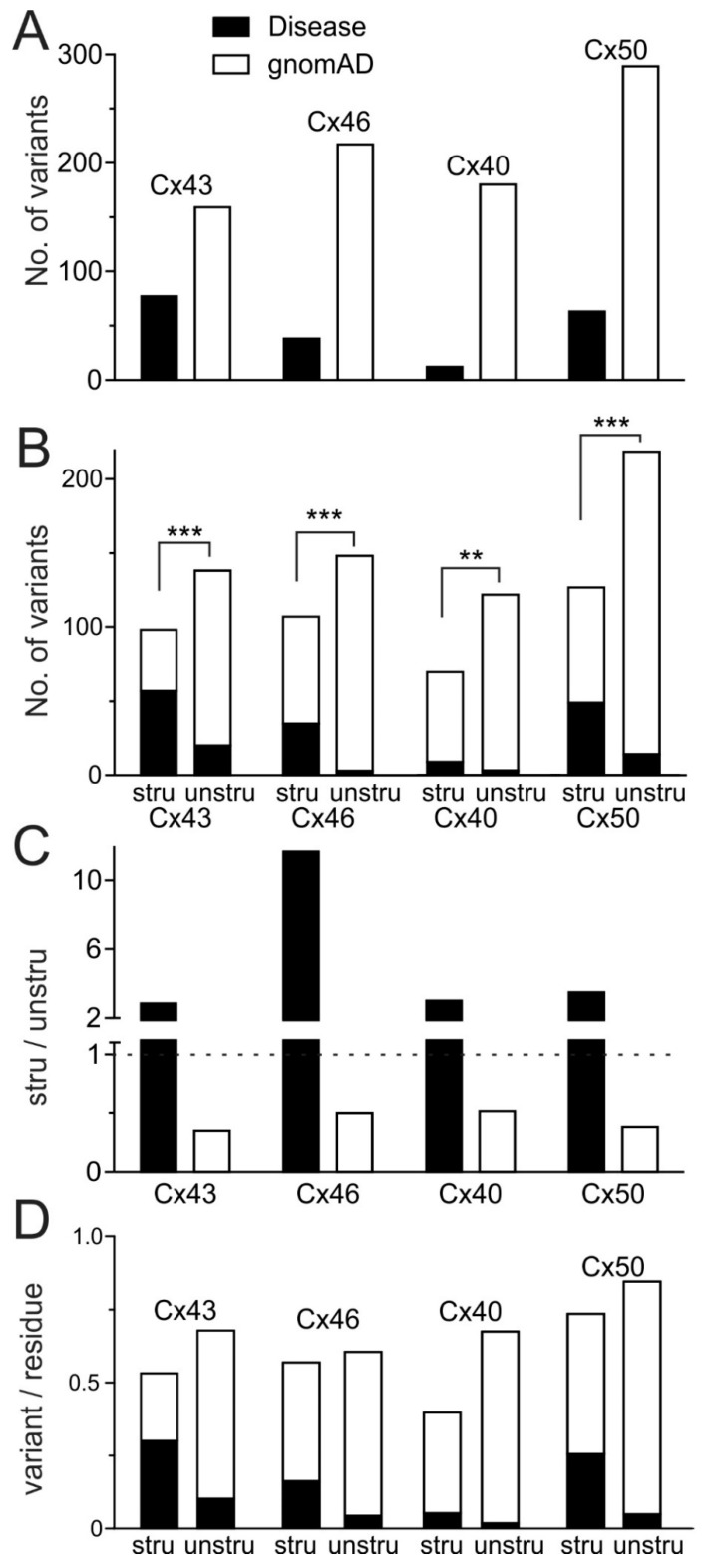
Disease-linked and gnomAD-linked missense variants showed unique pattern of distribution. (**A**) Total numbers of unique missense variants are plotted for each of the selected connexins for disease-linked (black bars) and gnomAD-linked (open bars). (**B**) The numbers of unique missense variants on structured domains (stru) vs. those on structure unresolved domains (unstru) are plotted as stacked bar graphs. The disease-linked variants showed a statistically significant different distribution from those gnomAD-linked variants on the structured and unstructured domains for each of these connexins (Fisher’s exact test ** *p* < 0.01 and *** *p* < 0.001). (**C**) The ratios of the variant numbers in the structured vs. unstructured domains (stru/unstru) are plotted as a bar graph. The disease-linked variants showed a two-fold or higher ratios, while the gnomAD-linked variants displayed consistent ratios of one half or lower for these connexins. (**D**) Stacked bars show the distribution of disease-linked (black) and gnomAD-linked (white) variants normalized by the length of structured or unstructured domains in each connexin.

To quantitatively analyze the relative distribution of disease-linked vs. gnomAD-linked missense variants for these connexins, we plotted the total number of unique missense variants of each connexin into a bar graph (Figure 5A) and then separated each bar into a stacked bar graph to show their distribution in structured or unstructured domains (Figure 5B). The absolute number of variants are quite different for each connexin, but a consistent and statistically significant pattern was revealed with the number of disease-linked variants more enriched in the structured domains than those on the unstructured domains and vice versa for gnomAD-linked variants (Figure 5B). The ratios of the number of missense variants in structured domains versus those in unstructured domains were plotted for each connexin. It is clear that disease-linked variants are consistently >2 fold more likely to be located in the structured domains, while the number of gnomAD-linked variants commonly show structured/unstructured ratios of <0.5 (Figure 5C). This reciprocal distribution pattern of disease versus gnomAD variants are consistent for all the connexins assessed (Figure 5C). To control for the possible contribution of protein length differences on the observed trends, we normalized the variant numbers to the number of residues in each domain (Figure 5D), and the general patterns of distribution were found to be similar to those shown in Figure 5B.

**Figure 6:**
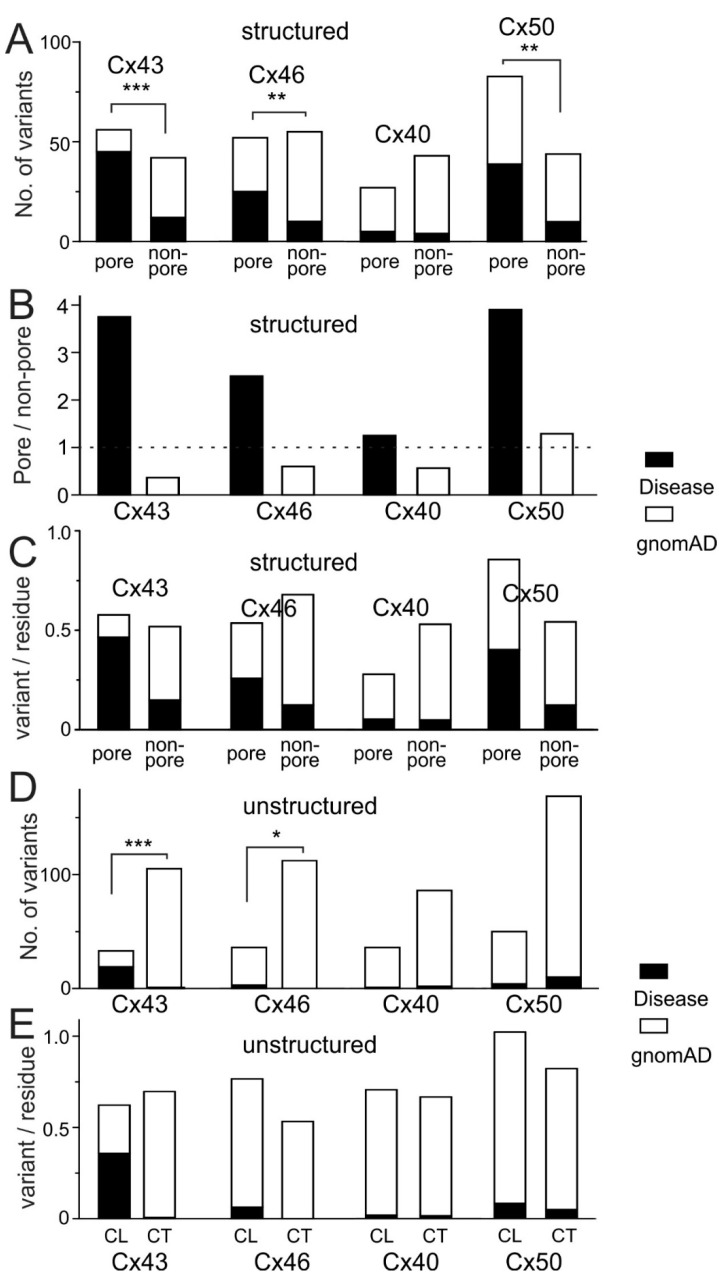
Disease-linked missense variants showed a preferential distribution in pore-lining domains and less likely to be in the carboxyl terminus (CT) domain. (**A**) Disease-linked variants (black part of each stacked bars) showed statistically significant differential distribution from those of gnomAD-linked variants (white part of each stacked bars) in these connexins (*** *p* < 0.001, ** *p* < 0.01), except Cx40. (**B**) Ratio (pore/non-pore) for disease-linked and gnomAD-linked missense variants are plotted for each of these connexins. The disease-linked variants showed a preferential localization on the pore domains than in non-pore domains, while no consistent pattern of distribution was observed for gnomAD-linked variants. Dotted line indicates the level of no preferential distribution between the pore and non-pore domains. (**C**) Stacked bars show the distribution of disease-linked (black) and gnomAD-linked (white) variants normalized by the length of pore and non-pore domains in each connexin. (**D**) Stacked bars illustrate the distributions of disease-linked (black) and gnomAD-linked (white) variants in the unstructured domains (CL and CT) for these connexins. Only Cx43 and Cx46 variants showed differential distribution (* *p* < 0.05, and *** *p* < 0.001). At the CT domain, gnomAD-linked variants dominated in all these connexins. (**E**) Stacked bars show the distribution of disease-linked (black) and gnomAD-linked (white) variants normalized by the length of CL and CT domains in each connexin.

Further dividing the structured domains into the pore (including NT, M1, E1, and M2 domains) and the non-pore domain (including M3, E2, and M4 domains), we found a statistically significant differential distribution of disease-linked and gnomAD-linked missense variants for these connexins, except for Cx40 (Figure 6A). The disease-linked variants were consistently more likely located on the pore domain than on the non-pore domain for these connexins (Figure 6B), while gnomAD-linked variants did not show a consistent pattern of distribution (Figure 6B). A similar pattern of distribution was observed when the variant number was normalized to the number of residues in these domains (Figure 6C). Dividing the unstructured domain into CL and CT, we plotted the distribution of disease- and gnomAD-linked variants in these two domains. As shown in Figure 6D, no consistent differential distributions were observed. The only reliable distribution pattern in these unstructured domains was that gnomAD-linked variants dominated in the CT domain (94% for Cx50, 98% for Cx40, 99% for Cx43, and 100% for Cx46), while few disease-linked variants were located in this domain (Figure 6D), indicating that the CT domain is better able to tolerate missense variations in these connexins. Again, a similar pattern of distribution was observed when the variant number was normalized to the number of residues in these domains (Figure 6E).

**Figure 7:**
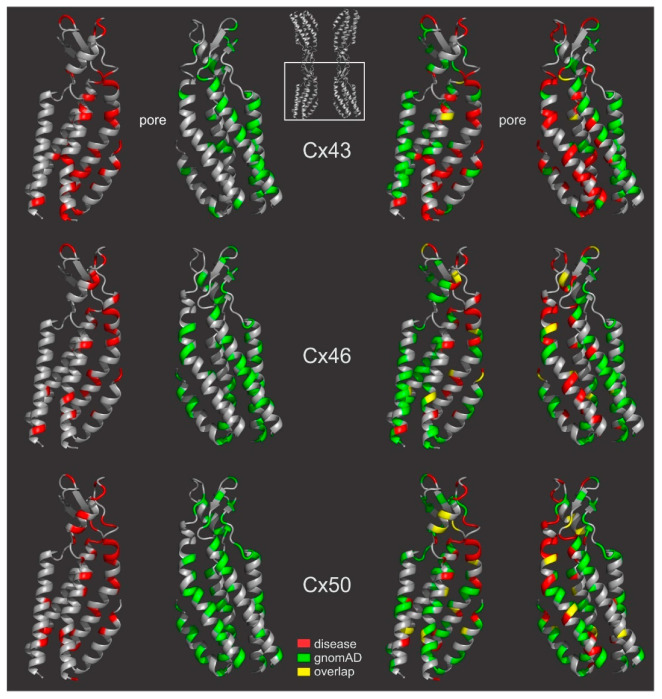
Disease- and gnomAD-linked missense variants are plotted on the gap junction structure. Homology structure models were developed for these connexins based on the Cx46 GJ structure (6MHQ) and the structure of the bottom two connexins as indicated are shown in cartoon view in the left panels to display the residues linking to diseases (red) or gnomAD (green) missense variant residue positions. Superimposed with both types of variants are shown on the right panels (overlapped residues are shown in yellow). A movie was generated to show these variants on 3D structure models (see supplementary movie).

To visualize the residue locations in the structured domains for disease- and gnomAD-linked missense variants in structure models, we colored the disease-linked residue positions in red and the gnomAD-linked residue positions in green in generated GJ homology structure models. These structure images show their localizations separately (Figure 7 left panels) and simultaneously on the backbone cartoon representations (Figure 7 right panels), highlighting the enrichment of the disease-linked missense variants in the pore domain and gnomAD-linked variants peripheral to the pore region (also see Supplementary movie).

### 2.4. Conservation Analysis on Connexins from Different Species

**Figure 8:**
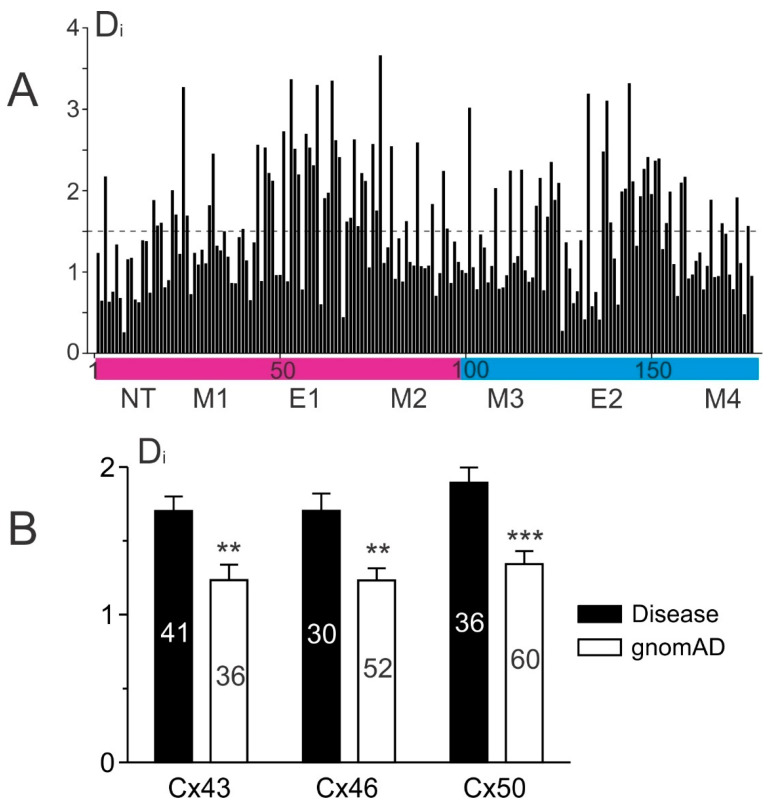
Disease-linked missense variants are enriched on highly conserved positions in connexins. (**A**) Over 9000 connexins from different species are aligned to sheep Cx46 on the structure resolved domains. The conservation of each residue position was plotted as a bar graph of the calculated Kullback–Leibler relative entropy (Di values). The dashed line indicates the average Di of these positions. Residues that fall into the magenta-colored bar are the pore domains including NT, M1, E1, and M2. Residues that fall into the blue-colored bar are the non-pore domains, including M3, E2, and M4 domains. (**B**) The averaged Di values of disease-linked variant positions are significantly higher than those corresponding to the gnomAD-linked variants for each of the selected connexin (mean ± SEM, ** *p* < 0.01 and *** *p* < 0.001). The total number of positions for each case are shown on the bar.

To study the residue position-dependent conservation for connexins from different species, we first collected 9201 connexin protein sequences homologous to human connexins and aligned them with sheep Cx46. Conservation scores (Di also known as the Kullback–Lerbler relative entropy) for each residue position was calculated as described [23] and displayed in Figure 8A. Residues on the structurally resolved domains are highly conserved throughout evolution with an average Di = 1.51 (dashed line in Figure 8A). Within these domains, the disease-linked variants are more likely to occur on those highly conserved residues with Di values significantly higher than those of gnomAD-linked variants in these three α connexins (Figure 8B). These data indicate that the highly conserved residues throughout evolution are important for biological function, and mutations on these highly conserved residues are often deleterious and linked to diseases.

### 2.5. Python-Based Statistical Coupling Analysis (pySCA) on Connexins

**Figure 9:**
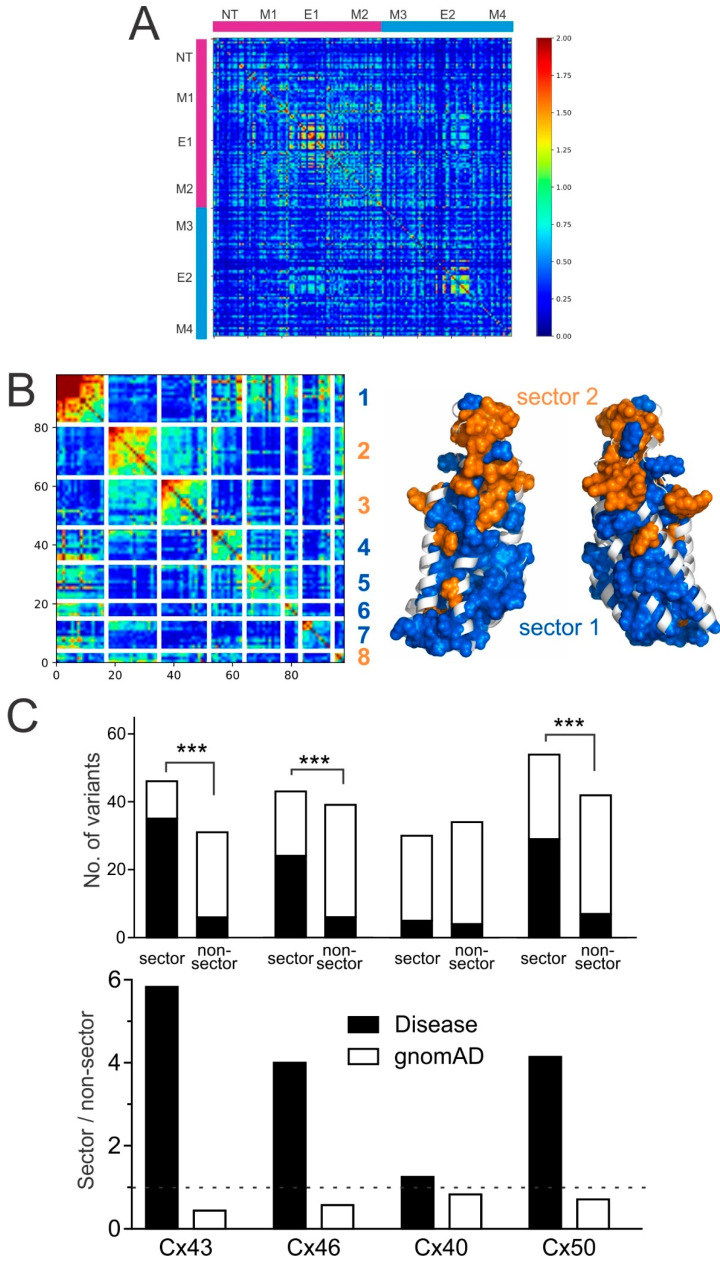
Statistical coupling analysis of connexin sequences. (**A**) Python-based statistical coupling analysis (pySCA) was used to obtain this evolutionary co-variation matrix on structured domains (based on Cx46 structure 6MHQ). The magenta-colored bars on the top and left illustrate the pore domain (NT, M1, E1, and M2), and similarly, the blue bars are the non-pore domain (M3, E2, and M4). The colored bar on the right indicates the degree of covariance within the conservation-weighted covariance matrix. Blue indicates no (or low) covariation, while red indicates strong (or high) covariation between two residues. (**B**) pySCA analysis revealed eight independent components (IC1–IC8, left panel) that belong to two protein sectors (as indicated by colored numbers). Sector 1 (blue) and sector 2 (orange) are shown on the gap junction structure (right panel, only two subunits are shown). The residues in each of the independent component are listed here: IC1 4 + 18 + 31 + 32 + 41 + 42 + 45 + 70 + 72 + 90 + 95 + 142 + 168 + 184 + 190 + 200 + 218; IC2 47 + 48 + 52 + 54 + 56 + 58 + 59 + 60 + 61 + 64 + 65 + 66 + 67 + 71 + 73 + 76 + 78 + 88; IC3 49 + 146 + 165 + 169 + 170 + 178 + 182 + 183 + 187 + 188 + 189 + 192 + 193 + 194 + 195 + 196 + 197; IC4 30 +40 + 63 + 80 + 81 + 85 + 96 + 145 + 153 + 204 + 214 + 215; IC5 2 + 15 + 77 + 79 + 82 + 92 + 143 + 144 + 157+ 163 + 164 + 207 + 211; IC6 6 + 17 + 69 + 150 + 173 + 198; IC7 7 + 10 + 11 + 14 + 19 + 22 + 23 + 25 + 26 + 28 + 33; IC8 55 + 74 + 160 + 203. (**C**) Stacked bars depicting disease-linked (black) and gnomAD-linked (white) missense variants are differentially distributed in protein sector and non-sector residue positions in these connexins except for Cx40. The ratios (sector/non-sector) of disease-linked (black bars) and gnomAD-linked (white bars) missense variants are plotted. Disease-linked variants in Cx43, Cx46, and Cx50 showed a preferential distribution on residue positions belonging to protein sectors, compared to gnomAD-linked variants (*** *p* < 0.001). Such a preferential distribution was not observed on variants in Cx40. The dotted line indicates the level of no preferential distribution between the protein sector and non-sector positions.

In addition to the analysis of primary sequence conservation in connexins from different species, some residue positions form a network of interactions to carry out a specific connexin function. Such networked residues are expected to display a high degree of co-variation among many connexins during evolution. To analyze the evolutionary co-variations in our collected connexins, we used the Python-based statistical coupling analysis (pySCA) package [20,23]. This program generated the co-variation matrix of the structure-resolved connexin residue positions (Figure 9A). Subsequently, spectral analysis was applied to the co-variation matrix to extract eigenmodes corresponding to top co-varying residue positions. Eight top eigenmodes were identified and were transformed into independent components (IC1–IC8, Figure 9B). IC1 showed the highest strength of internal correlations for residue positions within an IC and strong external correlations with IC4, IC5, IC6, and IC7. In contrast, IC1 showed much weaker external correlations with IC2, IC3, and IC8, while IC2, IC3, and IC8 showed strong correlations to one another. Based on the inter-IC correlation pattern, the eight ICs were grouped into two protein sectors, as shown in Figure 9B. Sector 1 (IC1, 4, 5, 6, and 7) maps to network residues at the interfaces of intra-subunit and inter-subunits, likely serving a role of connexin subunit folding and inter-subunit interaction, while sector 2 (IC2, 3, and 8) residues are clustered at the E1 and E2 domain and probably play a role in docking between two hemichannels. Interestingly, we observed a statistically significant differential distribution of the disease-linked and gnomAD-linked variants in the protein sector and non-sector residue positions in these connexins except for Cx40 (Figure 9C). The number of disease-linked variants on protein sector positions were consistently higher than those on non-sector positions and the reverse was true for gnomAD-linked variants on those residues equivalent to the structure-resolved positions (Figure 9C). These results are consistent with the notion that the identified residue positions in these sectors are functionally important and less likely to tolerate variation.

## 3. Discussion

In the present study, we analyzed the domain distribution of the most prevalent and position-sensitive missense variants in four α connexins (Cx43, Cx46, Cx40, and Cx50), including those documented to associate with inherited diseases [24,25,26,27,28] and those found in the reference genome/exome database, gnomAD [13,14]. In gnomAD, synonymous variants had a higher allele frequency than that of missense but the total number of unique missense variants was consistently greater than synonymous variants in these connexins. The total number of unique missense variants differs (see Figure 2 and Table S5), but the domain distribution showed a consistent pattern with disease-linked variants that are more prevalent on structure-resolved (and presumably stably folded) domains and less abundant on the structure-unresolved (and presumably disordered and unstable) domains. Opposite to this distribution pattern, the gnomAD-linked missense variants are less prevalent on structure-resolved and more abundant on the structure-unresolved domains. Further dividing the structure-resolved domains into pore and non-pore domains and the structure-unresolved domains into CL and CT domains, we found that disease-linked missense variants showed a higher distribution within the pore domain and lower distribution within the CT domain, and reciprocal to these observations, gnomAD-linked missense variants displayed lower distribution within the pore domain (except Cx50) and higher probability within the CT domain. Collectively, these distribution patterns indicate that the structured domains, especially the pore domains, are less tolerant to missense variants, which is probably due to their importance for fundamental GJ structure and function, while the unstructured domains, especially the CT domain, are more tolerant to missense variants, which are less likely to be deleterious to GJ structure and function. Comparing the sequence aligned human connexin orthologs in the structure-resolved domains, we found that disease-linked, but not gnomAD-linked, missense variants were on residue positions with higher conservation through evolution and are more likely to occur at residue positions that show co-variation. These patterns of domain preference, conservation, and co-variation of disease- and gnomAD-linked variants help to reveal connexin domains and residues essential for function. Ultimately, this knowledge will be useful for classifying the pathogenicity of newly identified connexin missense variants. However, we note that some residues in the structurally unresolved domains including CT in these and other connexins could play and have been shown to play an important role in trafficking, recycling, interactions with structural and signaling proteins, phosphorylation, and/or other modifications that regulate the functions of their GJ channels [29,30,31]. Nevertheless, on average, these domains can tolerate a higher number of missense variants in humans.

### 3.1. Pathogenicity of Disease-Linked Connexin Variants

Thus far, over two hundred genetic variations in the four α connexin genes have been identified in individuals and/or family members with one of these connexin-linked diseases. In most heritable cases, the diseases are inherited in an autosomal dominant manner in family members and co-segregated with the variants in generations. There are 89, 46, 13, and 70 variants found in *GJA1* (Cx43), *GJA3* (Cx46), *GJA5* (Cx40), and *GJA8* (Cx50), respectively, compiled from many independent studies (see references in Tables S1–S4). Collectively, the inherited cases together with those sporadic cases provided excellent genetic evidence that these genes play a pathogenic role in the respective diseases [32]. Additionally, several independent lines of evidence are also accumulated to support an association of these genes with disease. (1) Many functional in vitro studies of selected disease-linked variants show either loss of function or gain of function in GJ channels and/or hemichannels [33,34,35,36,37,38,39,40,41,42], including several mouse models showing consistent phenotypes mimicking the human disease symptoms [43,44,45,46]. (2) The vast majority of disease-linked variants (97%) were not observed in gnomAD. Even in those overlapped residue positions, only seven variants (7/35) have an identical amino acid substitution in the analyzed connexins. (3) Some of these variants occur on known key residues critical for biological function, including the highly conserved triple disulfide bridges formed by cysteine residues in the structured E1 and E2 domains, which are necessary for connexin folding [47] and those docking-required hydrogen bond forming residues at the interface between two hemichannels [48,49,50]. (4) Bioinformatic software packages were developed recently to predict pathogenicity of rare coding variants. These prediction algorithms consider several metrics including amino acid residue and/or nucleotide evolutionary conservation, the residue location on structure models, biochemical properties of the amino acid substitutions, and the combination of these factors to award a predictive likelihood score for a variant in between pathogenic and benign [51,52,53]. These programs are now routinely used to classify newly identified connexin variants [54]. It is not surprising that most of the recently reported disease-linked connexin variants are predicted to be pathogenic by these in silico approaches [55,56,57,58].

All the genetic linkages and segregations in multiple families, functional in vivo and in vitro studies, in silico predictions, and lack of existence in the reference genome databases provide overwhelming evidence that these connexin genes do play a pathogenic role in their respective diseases. However, whether each specific disease-linked connexin variant plays a pathogenic role is often unclear. The clarity will depend on the accumulated evidence on that particular variant, including the number of independent cases, segregation in generations, the existence and abundance of the same variant in the reference genome, in silico predictions, and more importantly, functional studies in relevant cellular or animal models. The American College of Medical Genetics and Genomics/Association for Molecular Pathology (ACMG/AMP) has developed standards and guidelines for interpretation of sequence variants to classify variants into one of the following five categories: pathogenic, likely pathogenic, uncertain significance, likely benign, and benign [54]. Expert panels have started to gather available information to curate the disease-linked variants against the reference genome, gnomAD. Currently, most of the connexin variants described in this study have not been fully reviewed by expert panels. We hope our present work will help facilitate the variant classification process in several ways. First, we collated many original references/reports containing much of the clinical information on the genetics, segregation, allele frequency, ethnic origin, etc., needed by the panels. Second, we showed that gnomAD missense variants can be mapped on topological and structure models, and the detailed information on these variants (prevalence, population specific allele frequency, etc.) could be easily accessible on the gnomAD website (https://gnomad.broadinstitute.org/ accessed on 1 July 2021). Third, we revealed a remarkable segregation on spatial distribution, conservation, and co-variation between disease- and gnomAD-linked variants, which could further enrich and improve the accuracy of pre-existing in silico prediction algorithms for these connexins.

For those minority variant positions found in both the disease- and gnomAD-linked cohort (yellow residue positions in Figure 4 and Figure 7), ≈80% are different amino acids in the disease-linked compared to the gnomAD substitutions. We found only seven identical missense variants (with the same substitution) in these connexins in the disease and gnomAD cohort. Several possibilities could account for identical variants in the disease and gnomAD cohorts. (1) A small percentage of the disease-associated variants may not play a true pathogenic role and may be falsely associated with the disease in a “guilty by association” scenario. Often, but not always, the same variants could exist in gnomAD in a subpopulation at a high allele frequency, greater than disease prevalence in the general population. This possibility needs to be determined for each of these overlapped variants. One of the missense Cx50 variants, Asn220Asp, exists in non-Finnish Europeans at an allele frequency of 0.00424 in gnomAD, which is much higher than the prevalence (0.00072) for congenital cataracts [59], suggesting that this is likely a benign variant. (2) A disease-linked variant may be truly pathogenic, but it could still exist in the reference genome database due to lack of expressivity, which could be due to one or more of the following factors: incomplete penetrance, lack of preconditional environmental factors or comorbidities, late onset of disease phenotype, and/or other associated modifier genes. (3) Sequencing technology is not yet perfect in identifying variants. Both sequencing errors and annotation artefacts could occur and exist in both the disease or gnomAD cohort [14,60]. Follow-up studies are required to consolidate the variation or future improvement of sequencing technology is needed to eliminate or reduce the errors in sequencing and annotation.

### 3.2. Limitations of Our Analysis and Future Directions

Several Mendelian-inherited diseases are well established to be due to genetic variation in connexin genes [10]. Among the four α connexin–disease pairs discussed in this study, ODDD-linked *GJA1* (Cx43) variants were not the first discovered but have revealed the largest number of disease-linked variants among these four α connexins. Atrial fibrillation (AF)-linked *GJA5* (Cx40) variants showed the lowest number of variants among these α connexins. The limited number of variants of *GJA5* (Cx40) reduced the power of their domain or subdomain distribution analysis, although we observed a similar pattern of larger domain distribution as those from the other three α connexins. As the number of missense variants is low, the distribution representation in topological and structure models were not included in this study. Future genetic studies may find more AF-linked *GJA5* (Cx40) variants, providing a better domain representation.

Novel connexin gene-disease pairs have recently been reported on connexins that had not been previously linked to any inherited disease. For example, two variants in *GJC1* (Cx45) were recently found to associate with inherited atrial conduction defects in an autosomal dominant manner [61,62]. In addition, some connexin gene variants that have been linked to one disease could have a subset of variants linked to one or more related or unrelated inherited diseases. A well-known example is neurosensory hearing loss linked variants in *GJB2* (Cx26) with a vast majority of cases inherited in an autosomal recessive manner. However, a subset of *GJB2* (Cx26) variants is not only associated with hearing loss but also with severe skin diseases; moreover, these *GJB2* variants are inherited autosomal dominantly and classified as syndromic hearing loss [9,63]. Another example is *GJA1* (Cx43). Variants in this gene have also been reported to be linked to other ODDD-related or unrelated diseases (see OMIM web https://omim.org/ accessed on 1 July 2021). It is worth noting that there are ten connexin genes, including three α connexin genes (*GJA4* (Cx37), *GJA9* (Cx59), *GJA10* (Cx62)), that have not yet been linked to any inherited diseases and require more studies to establish if they play a role in pathogenicity.

Here, we focused our analysis on genetic variants in the coding regions of connexin genes. Variants in the noncoding regions including 3′- and 5′-untranslated regions (UTRs) have key splice acceptor/donor sites known to be important for proper transcription. The intronic regulatory regions of these connexin genes could also play important roles in regulating transcription and affecting the biosynthesis of these connexins in tissue cells. Sequencing the entire gene and the gene regulatory regions of the genome should be included for future connexin disease-linked sequencing endeavors, which could help to reveal if variations in these regions play a role in connexin channelopathy.

## 4. Materials and Methods

### 4.1. Sequence Alignment of Human α Connexins

Human α connexin sequences were obtained from UniProt (https://www.uniprot.org/ accessed on 1 July 2021) [64], and they were aligned in ClustalOmega using default settings prior to visualization in Jalview (Version 2.11.1.4, the Barton Group, University of Dundee, Scotland, UK). After sequence alignment, the conservation scores were obtained for each residue position and plotted using Jalview [22].

### 4.2. Structure Models

Human Cx43, Cx46, and Cx50 topological models were developed after sequence alignment of these human connexins with that of sheep Cx46 to assign each residue position relative to the plasma membrane based on the cryo-EM structure of sheep Cx46 (6MHQ) [8]. Similarly, homology structure models of human Cx43, Cx46, and Cx50 were generated in Modeller (Version 9.17, Andrej Sali Lab, San Francisco, CA, USA) based on aligned sequences of these connexins against the sheep Cx46 [65]. The cartoon representations of the backbone structure with variants were visualized, and images and the supplemental movie were created using PyMOL (The PyMOL Molecular Graphics System, Version 2.4.0 Schrödinger, Inc., New York, NY, USA) [21].

### 4.3. Python-Based Statistical Coupling Analysis (pySCA) of Connexins from Different Species

From the National Center for Biotechnology Information protein database, we collected a total 9201 connexin sequences homologous to human connexins by their annotated names, such as *GJA1* (Cx43), *GJB2* (Cx26), *GJC1* (Cx45), etc. without any species restriction. Taxonomic annotations were not used and did not affect our analysis. *GJE1* (Cx23) was excluded because the protein sequences lacked the characteristically spaced triple cysteine residue signature known to be important for connexin folding and the formation of GJ channels. These sequences were aligned using ClustalOmega and visualized in Jalview with sheep Cx46 taken as the reference sequence and 6MHQ as the GJ reference structure [8]. Structurally unresolved domains were excluded in coupling analysis because these domains displayed large variations in both length and the amino acid sequence.

We used the Python-based statistical coupling analysis (pySCA) package as described earlier [20,23] to analyze groups of amino acid residue positions for evolutionary co-variation. pySCA is a well-established and validated bioinformatics approach, which is used to determine residue covariance within a protein family [20,66]. The pySCA scripts were run using a Linux system to obtain conservation scores (the Di values or Kullback–Lerbler relative entropy) and a co-variation matrix. The default values in pySCA were used for sequence filter settings with the exception that we increased the maximum fractional identity between query and reference sequences from 0.80 to 0.85 to include smaller variations between two closely related lens connexins, Cx46 and Cx50. After applying sequence weights, a total of 117 effective sequences were obtained in the alignment with an average pairwise identity of 0.427. Spectral analysis of the co-variation matrix was used to extract top eigenmodes corresponding to top co-varying residue positions. Significant eigenmodes were determined by comparing the spectral analysis of the co-variation matrix with 10 trials of randomized residue positions. Then, the top eigenmodes were transformed into independent components (ICs). Each IC contains residue positions that most strongly co-vary among themselves and most weakly co-vary with residue positions in other ICs. Residues in each IC were determined as the top 5% contributors to the cumulative density function. Eight total ICs were identified, which were further grouped into two protein sectors in a constructed submatrix based on the positional overlap between ICs.

### 4.4. Statistical Analysis

Bar graphs or stacked bar graphs were generated to show patterns of missense variant distribution on structured vs unstructured domains. An unpaired t-test was used to compare the Di values for disease- and gnomAD-linked missense variant residue positions. Fisher’s exact test (GraphPad 5, Version 5.04 GraphPad Software, Inc., San Diego, CA, USA) was used to compare the fractional distribution of disease- and gnomAD-linked variants among the selected portions of GJs: structured (or structure-resolved) vs. unstructured (or structure-unresolved) domains, pore vs. non-pore domains, or sector vs. non-sector residue positions.

## 5. Conclusions

Inherited disease-linked and the reference genome gnomAD-linked four α connexin missense variants displayed distinct distribution patterns with disease-linked but not gnomAD-linked variants more likely observed in well-ordered/structured domains, especially pore domains. On the other hand, the gnomAD-linked variants but not disease-linked variants are more likely observed in unstructured domains, especially the CT domain. Furthermore, within the structure-resolved domains, the disease-linked but not the gnomAD-linked variants are more likely on highly conserved residues and residues that show co-variation. Our findings provide a structure-based framework that will aid in classifying the pathogenicity of newly identified disease-linked variants.

## Data Availability

Data in our manuscript are available on request.

## Supplementary Materials

The following are available online at https://www.mdpi.com/article/10.3390/ijms22157832/s1, Table S1: Oculodentodigital dysplasia (ODDD)-linked Cx43 (GJA1) variants; Table S2: Cataract linked Cx46 (GJA3) variants; Table S3: Atrial fibrillation (AF)-linked Cx40 (GJA5) variants; Table S4: Cataract-linked Cx50 (GJA8) variants; Table S5: Summary of different types of variants. Supplementary movie: Structural localizations of disease- and gnomAD-linked missense variants.
