# Supplementary material for: Differential Domain Distribution of gnomAD- and Disease-Linked Connexin Missense Variants
Source: Int J Mol Sci. 2021 Jul 22;22(15):7832. doi: 10.3390/ijms22157832 (PMC8346055; doi:10.3390/ijms22157832)
Supplement: Supplementary file 1 [file ijms-22-07832-s001.zip › ijms-1281081 supplemental-tables-June10-2021.pdf]

Table S1. Oculodentodigital dysplasia (ODDD)-linked Cx43 (*GJA1*) variants

No.	Mutation (protein)	Location	Type of Mutation	Inherit.	References
1	Gly2Val	NT	Missense	AD	[1]
2	Gly2fsTer7	NT	Frame shift	compound heterozygous with Arg101Ter	[2]
3	Asp3Asn	NT	Missense	AD	[3, 4]
4	Ser5Cys	NT	Missense	AD	[5]
5	Leu7Val	NT	Missense	AD	[4]
6	Leu11Ile	NT	Missense	AD	[6]
7	Leu11Phe	NT	Missense	AD	[7, 8]
8	Leu11Pro	NT	Missense	AD	[4, 9]
9	Tyr17Ser	NT	Missense	AD	[10]
10	Ser18Pro	NT	Missense	AD	[10]
11	Gly21Arg	M1	Missense	AD	[10]
12	Gly22Glu	M1	Missense	AD	[4, 10-12]
13	Gly22Arg	M1	Missense	AD	[4]
14	Lys23Thr	M1	Missense	AD	[3, 10]
15	Trp25Cys	M1	Missense	AD	[13, 14]
16	Leu26Pro	M1	Missense	AD	[15]
17	Ser27Pro	M1	Missense	AD	[16]
18	Ile31Met	M1	Missense	AD	[16]
19	Ile31Phe	M1	Missense	AD	[17]
20	Phe32Val	M1	Missense	AD	[18]
21	Arg33Ter	M1	Stop gained	AR	[19]
22	Ala40Val	M1	Missense	AD	[10, 16, 20, 21]

23	Val41Leu	M1	Missense	AD	[22]
24	Val41_Ala44 del	M1	Inframe deletion	AD	[23]
25	Glu42Gln	M1	Missense	AD	[24]
26	Asp47His	E1	Missense	AD	[25]
27	Glu48Lys	E1	Missense	AD	[26]
28	Gln49dup	E1	Inframe insertion	AD	[4]
29	Gln49Glu	E1	Missense	AD	[27]
30	Gln49Lys	E1	Missense	AD	[10]
31	Gln49Pro	E1	Missense	AD	[4]
32	Phe52dup	E1	Inframe insertion	AD	[10]
33	Asn55Asp	E1	Missense	AD	[4]
34	Gln57SerfsTer6	E1	Frame shift	AR	[28]
35	Gln58His	E1	Missense	AD	[4]
36	Pro59Ala	E1	Missense	AD	[4]
37	Pro59His	E1	Missense	AD	[29]
38	Ser69Tyr	E1	Missense	AD	[4, 16]
39	His74Leu	M2	Missense	AD	[4]
40	His74Pro	M2	Missense	AD	[30]
41	Arg76Ser	M2	Missense	AD	[10]
42	Arg76Cys	M2	Missense	AD	[27]
43	Arg76His	M2	Missense	AR	[31]
44	Val85Met	M2	Missense	AD	[3]
45	Ser86Tyr	M2	Missense	AD	[25]
46	Leu90Val	M2	Missense	AD	[10]
47	His95Arg	M2	Missense	AD	[4, 32]

48	Val96Ala	M2	Missense	AD	[4]
49	Val96Gly	M2	Missense	AD	[15]
50	Val96Met	M2	Missense	AD	[33]
51	Val96Glu	M2	Missense	AD	[34]
52	Tyr98Cys	CL	Missense	AD	[10]
53	Arg101Ter	CL	Stop gained	AR, compound heterozygous with Gly2fsTer7	[2]
54	Lys102Asn	CL	Missense	AD	[10]
55	Leu106Pro	CL	Missense	AD	[4]
56	Leu106Arg	CL	Missense	AD	[25]
57	Glu110Asp	CL	Missense	AD	[20]
58	Leu113Pro	CL	Missense	AD	[16] [34]
59	Ile130Thr	CL	Missense	AD	[10]
60	Lys134Glu	CL	Missense	AD	[10]
61	Lys134Asn	CL	Missense	AD	[16]
62	Lys134del	CL	Inframe deletion	AD	[18]
63	Gly138Asp	CL	Missense	AD	[3, 35]
64	Gly138Arg	CL	Missense	AD	[10]
65	Gly138Ser	CL	Missense	AD	[3, 16, 36]
66	Gly143Asp	CL	Missense	AD	[3]
67	Gly143Ser	CL	Missense	AD	[16]
68	Lys144Glu	CL	Missense	AD	[4]
69	Val145Gly	CL	Missense	AD	[4]
70	Met147Thr	CL	Missense	AD	[20]
71	Arg148Gly	CL	Missense	AD	[4]

72	Arg148Gln	CL	Missense	AD	[3, 4, 16]
73	Arg148Ter	Cl	Stop gained	AR	[37]
74	Thr154Ala	M3	Missense	AD	[38]
75	Thr154Asn	M3	Missense	AD	[34]
76	Thr154Ile	M3	Missense	AD	[18]
77	Phe169del	M3	Inframe deletion	AD	[20]
78	Pro193Leu	E2	Missense	AD	[4]
79	His194Pro	E2	Missense	AD	[39]
80	Ser201Phe	E2	Missense	AD	[4]
81	Ser201Tyr	E2	Missense	AD	[3]
82	Arg202His	E2	Missense	AD	[3, 10, 16]
83	Lys206Arg	M4	Missense	AD	[40]
84	Phe212Ile	M4	Missense	AD	[41]
85	Val216Leu	M4	Missense	AD	[10]
86	Ser220Tyr	M4	Missense	AD	[34]
87	Tyr230fs Ter236	M4	Frame shift	AD	[13, 42]
88	Arg239Gln	CT	Missense	AR	[43]
89	Cys260fs Ter307	CT	Frame shift	AD	[44]

Abbreviations: AD, autosomal dominant inheritance; AR, autosomal recessive inheritance; NT, amino terminal domain; M1-4, transmembrane domain 1-4; E1, the first extracellular domain; E2, the second extracellular domain; CL, cytoplasmic loop; CT, carboxyl terminal domain; ODDD, oculodentodigital dysplasia; inherit., inheritance.

An Excel format of this table is available on request.

Table S2. Cataract linked Cx46 (*GJA3*) variants

No.	Mutation (protein)	Location	Type of Mutation	Inherit.	Reference and notes
1	Met1Val	NT	Start lost	AD	[45]
2	Gly2Asp	NT	Missense	AD	[46]
3	Asp3His	NT	Missense	AD	[47]
4	Asp3Tyr	NT	Missense	AD	[48, 49]
5	Leu11Ser	NT	Missense	AD	[50, 51]
6	Thr19Met	NT	Missense	AD	[52, 53]
7	Gly22Ser	M1	Missense	AD	[54]
8	Val28Leu	M1	Missense	AD	[55]
9	Val28Met	M1	Missense	AD, incomplete	[56]
10	Ile31Asn	M1	Missense	AD	[57]
11	Phe32Leu	M1	Missense	AD	[58]
12	Arg33Leu	M1	Missense	AD	[59]
13	Glu42Ala	M1	Missense	AD	[60]
14	Val44Met	M1	Missense	AD	[61-63]
15	Trp45Ser	M1	Missense	AD	[64]
16	Asp47Asn	E1	Missense	AD	[65, 66]
17	Glu48Gly	E1	Missense	AD	[67]
18	Ser50Pro	E1	Missense	AD	[68]
19	Asn55Asp	E1	Missense	AD	[69]
20	Pro59Leu	E1	Missense	AD	[47, 70-73]
21	Glu62Lys	E1	Missense	AD	[68]
22	Asn63Ser	E1	Missense	AD	[74]

23	Arg76Gly	E1	Missense	AD	[56]
24	Arg76His	E1	Missense	AD, incomplete	[56, 72, 75]
25	Thr87Met	M2	Missense	AD	[47, 76]
26	Leu90Phe	M2	Missense	AD	[60]
27	Val139Met	CL	Missense	AD	[77]
28	Gly143Arg	M3	Missense	AD	[78, 79]
29	Gly143Glu	M3	Missense	AD	[80]
30	Thr148Ile	M3	Missense	AD	[81]
31	Lys156Gln	M3	Missense	AD	[53]
32	Pro187Leu	E2	Missense	AD	[82]
33	Pro187Ser	E2	Missense	AD	[83]
34	Asn188Thr	E2	Missense	AD	[84]
35	Asn188Ile	E2	Missense	AD	[85]
36	Phe193Ser	E2	Missense	AD	[86]
37	Pro197Ser	M4	Missense	AD	[87]
38	Glu199Ala	M4	Missense	AD	[68]
39	Phe206Ile	M4	Missense	AD	[88]
40	Ser258GlnfsTer68	CT	Frame shift	AD	[55]
41	His318ProfsTer8	CT	Frame shift	AR	[89]
42	Ser380GlnfsTer87	CT	Frame shift	AD	[74]
43	Ser381ArgfsTer48	CT	Frame shift	AD	[73]
44	Ser385GlufsTer83	CT	Frame shift	AD	[90]
45	Ala397GlyfsTer71	CT	Frame shift	AD	[91]
46	Thr400HisfsTer31	CT	Frame shift	AD	[92]

Abbreviations: AD, autosomal dominant inheritance; AR, autosomal recessive inheritance; NT, amino terminal domain; M1-4, transmembrane domain 1-4; E1, the first extracellular domain; E2, the second extracellular domain; CL, cytoplasmic loop; CT, carboxyl terminal domain; ODDD, oculodentodigital dysplasia; inherit., inheritance.

An Excel format of this table is available on request.

Table S3. Atrial fibrillation (AF)-linked Cx40 (*GJA5*) variants

No.	Mutation (protein)	Location	Type of Mutation	Inherit.	Reference and notes
1	Gly38Asp	M1	Missense	somatic	[93]
2	Gln49Ter	E1	Stop gained	AD	[94]
3	Iso75Phe	M2	Missense	AD	[95]
4	Val85Iso	M2	Missense	AD	[96]
5	Pro88Ser	M2	Missense	somatic	[93]
6	Ala96Ser	M2	Missense	AD	[93, 97, 98]
7	Lys107Arg	CL	Missense	AD	[99]
8	Met163Val	M3	Missense	somatic	[93]
9	Leu221Iso	M4	Missense	AD	[96]
10	Leu223Met	M4	Missense	AD	[99]
11	Leu229Met	M4	Missense	AD	[96]
12	Gln236His	CT	Missense	AD	[99]
13	Iso257Leu	CT	Missense	AD	[99]

Abbreviations: AD, autosomal dominant inheritance; AR, autosomal recessive inheritance; AF, atrial fibrillation; NT, amino terminal domain; M1-4, transmembrane domain 1-4; E1, the first extracellular domain; E2, the second extracellular domain; CL, cytoplasmic loop; CT, carboxyl terminal domain; inherit., inheritance.

An Excel format of this table is available on request.

Table S4. Cataract-linked Cx50 (*GJA8*) variants

No.	Mutation (protein)	Location	Type of Mutation	Inherit.	Reference and notes
1	Trp4Arg	NT	Missense	AD	[100]
2	Leu7Pro	NT	Missense	AD	[101, 102]
3	Gly8_Leu11del	NT	Inframe deletion	AD	[103]
4	Ser18Phe	NT	Missense	AD	[89]
5	Gly22Ser	M1	Missense	AD	[54]
6	Arg23Thr	M1	Missense	AD	[104]
7	Trp25Ala	M1	Missense	AD	[53]
8	Ile31Thr	M1	Missense	AD	[105]
9	Ile31HisfsX18	M1	Frame shift	AR	[47]
10	Phe32Ile	M1	Missense	AD	[106, 107]
11	Thr39Arg	M1	Missense	AD	[108, 109]
12	Ala40Val	M1	Missense	AD	[47]
13	Val44Ala	M1	Missense	AD	[110, 111]
14	Val44Glu	M1	Missense	AD	[112]
15	Val44Met	M1	Missense	AD	[63, 113]
16	Trp45Ser	M1	Missense	AD	[47, 53, 109, 114]
17	Trp45Leu	M1	Missense	AD	[109, 113]
18	Trp45Arg	M1	Missense	AD	[115]
19	Gly46Val	M1	Missense	AD	[116]
20	Gly46Arg	M1	Missense	AD	[108]
21	Asp47Asn	E1	Missense	AD	[117-119]
22	Asp47His	E1	Missense	AD	[120, 121]
23	Asp47Tyr	E1	Missense	AD	[122]
24	Glu48Lys	E1	Missense	AD	[123]
25	Asp51Asn	E1	Missense	AD	[47, 109]
26	Phe52Leu	E1	Missense	AD	[90]
27	Thr56Pro	E1	Missense	AD	[124]

28	Pro59Ala	E1	Missense	AD	[89, 125]
29	Gly60Arg	E1	Missense	AD	[55]
30	Val64Gly	E1	Missense	AD	[64]
31	Asp67Gly	E1	Missense	AD	[126]
32	Phe70Leu	E1	Missense	AD	[109]
33	Ser73Phe	E1	Missense	AD	[60]
34	Ser73Pro	E1	Missense	AD	[72, 127]
35	Arg76His	M2	Missense	AD	[125, 128]
36	Arg76Cys	M2	Missense	AD	[126]
37	Val79Leu	M2	Missense	AD	[129]
38	Pro88Gln	M2	Missense	AD	[130]
39	Pro88Ser	M2	Missense	AD	[55, 131]
40	Pro88Thr	M2	Missense	AD	[132]
41	His95_Ala96InsYAVHY	M2	Inframe insertion	AD	[133]
42	Val97Gly	M2	Missense	AD	[109]
43	His98Pro	CL	Missense	AD	[101]
44	His98Arg	CL	Missense	AD	[68]
45	Arg101Leu	CL	Missense	AD	[113]
46	Leu143_Leu147del	CL	Inframe deletion	AD	[134]
47	Gly145Trp	CL	Missense	AD	[106]
48	His154Asp	M3	Missense	AD	[135]
49	Glu162Lys	M3	Missense	AD	[53]
50	Pro189Leu	E2	Missense	AD	[136]
51	Pro189Ser	E2	Missense	AD	[137]
52	Val196Met	E2	Missense	AR	[87]
53	Arg198Gln	E2	Missense	AD	[112, 138]
54	Arg198Trp	E2	Missense	AD	[139]
55	Pro199Ser	E2	Missense	AD	[87]
56	Glu201Lys	E2	Missense	AD	[140]
57	Thr203AsnfsX47	E2	Frameshift	AR	[141]

58	Asn220Asp	M4	Missense	AD, incomplete	[109, 142]
59	Ile247Met	CT	Missense	AD	[143, 144]
60	Ala256GlyfsX123	CT	Frameshift	AR	[145]
61	Ser258Phe	CT	Missense	AD	[146]
62	Ser259Tyr	CT	Missense	AD	[72]
63	Val275Ile	CT	Missense	AD	[77]
64	Ser276Phe	CT	Missense	AD	[147]
65	His277Tyr	CT	Missense	Cataract	[148]
66	Pro280Arg	CT	Missense	AD	[102]
67	Leu281Cys	CT	Missense	Cataract	[149]
68	Leu292Gln	CT	Missense	Sporadic, AD	[109]
69	Glu368Gln	CT	Missense	AD	[150]
70	Arg425Ter	CT	Stop gained	Sporadic, AD	[68]

Abbreviations: AD, autosomal dominant inheritance; AR, autosomal recessive inheritance; NT, amino terminal domain; M1-4, transmembrane domain 1-4; E1, the first extracellular domain; E2, the second extracellular domain; CL, cytoplasmic loop; CT, carboxyl terminal domain; ODDD, oculodentodigital dysplasia; inherit., inheritance.

An Excel format of this table is available on request.

Table S5. Summary of different types of variants.

Disease-linked variants									
Gene (protein)	missense	Synonymous	stop gained	frame shift	inframe deletion	inframe insertion	stop lost	start lost	**Total**
GJA1 (Cx43)	77		3	4	3	2			**89**
GJA3 (Cx46)	38			7				1	**46**
GJA5 (Cx40)	12		1						**13**
GJA8 (Cx50)	63		1	3	2	1			**70**
**Total**	**190**		**5**	**14**	**5**	**3**		**1**	**218**

gnomAD-linked variants									
Gene (protein)	missense	synonymous	stop gained	frame shift	inframe deletion	inframe insertion	stop lost	start lost	**Total**
GJA1 (Cx43)	159	118	5	4	2	1	1		**290**
GJA3 (Cx46)	217	140	6	11	9	5			**388**
GJA5 (Cx40)	180	92	7	7	2	1	1	1	**291**
GJA8 (Cx50)	289	153	7	9	10	1	1		**470**
**Total**	**845**	**503**	**25**	**31**	**23**	**8**	**3**	**1**	**1439**

Detailed information on the references and the nature of variation of disease-linked variants are in Table 1-4. The gnomAD-linked variants are obtained from website (https://gnomad.broadinstitute.org/).

References

1. de la Parra, D. R.; Zenteno, J. C., A new GJA1 (connexin 43) mutation causing oculodentodigital dysplasia associated to uncommon features. *Ophthalmic Genet.* **2007,** 28, (4), 198-202.
2. Jamsheer, A.; Badura-Stronka, M.; Sowinska, A.; Debicki, S.; Kiryluk, K.; Latos-Bielenska, A., A severe progressive oculodentodigital dysplasia due to compound heterozygous GJA1 mutation. *Clin. Genet.* **2010,** 78, (1), 94-7.
3. Fenwick, A.; Richardson, R. J.; Butterworth, J.; Barron, M. J.; Dixon, M. J., Novel Mutations in GJA1 Cause Oculodentodigital Syndrome. *J. Dent. Res.* **2008,** 87, (11), 1021-1026.
4. Paznekas, W. A.; Karczeski, B.; Vermeer, S.; Lowry, R. B.; Delatycki, M.; Laurence, F.; Koivisto, P. A.; Van Maldergem, L.; Boyadjiev, S. A.; Bodurtha, J. N.; Jabs, E. W., GJA1 mutations, variants, and connexin 43 dysfunction as it relates to the oculodentodigital dysplasia phenotype. *Hum. Mutat.* **2009,** 30, (5), 724-33.
5. Himi, M.; Fujimaki, T.; Yokoyama, T.; Fujiki, K.; Takizawa, T.; Murakami, A., A case of oculodentodigital dysplasia syndrome with novel GJA1 gene mutation. *Jpn. J. Ophthalmol.* **2009,** 53, (5), 541-5.
6. Porntaveetus, T.; Srichomthong, C.; Ohazama, A.; Suphapeetiporn, K.; Shotelersuk, V., A novel GJA1 mutation in oculodentodigital dysplasia with extensive loss of enamel. *Oral Dis.* **2017,** 23, (6), 795-800.
7. Liu, X. Z.; Xia, X. J.; Adams, J.; Chen, Z. Y.; Welch, K. O.; Tekin, M.; Ouyang, X. M.; Kristiansen, A.; Pandya, A.; Balkany, T.; Arnos, K. S.; Nance, W. E., Mutations in GJA1 (connexin 43) are associated with non-syndromic autosomal recessive deafness. *Hum. Mol. Genet.* **2001,** 10, (25), 2945-51.
8. Jamsheer, A.; Wisniewska, M.; Szpak, A.; Bugaj, G.; Krawczynski, M. R.; Budny, B.; Wawrocka, A.; Latos-Bielenska, A., A novel GJA1 missense mutation in a Polish child with oculodentodigital dysplasia. *J Appl Genet* **2009,** 50, (3), 297-9.
9. Kelly, S. C.; Ratajczak, P.; Keller, M.; Purcell, S. M.; Griffin, T.; Richard, G., A novel GJA 1 mutation in oculo-dento-digital dysplasia with curly hair and hyperkeratosis. *Eur. J. Dermatol.* **2006,** 16, (3), 241-5.
10. Paznekas, W. A.; Boyadjiev, S. A.; Shapiro, R. E.; Daniels, O.; Wollnik, B.; Keegan, C. E.; Innis, J. W.; Dinulos, M. B.; Christian, C.; Hannibal, M. C.; Jabs, E. W., Connexin 43 (GJA1) Mutations Cause the Pleiotropic Phenotype of Oculodentodigital Dysplasia. *The American Journal of Human Genetics* **2003,** 72, (2), 408-418.
11. Gumus, E., A rare symptom of a very rare disease: a case report of a oculodentodigital dysplasia with lymphedema. *Clin. Dysmorphol.* **2018,** 27, (3), 91-93.
12. Kumar, V.; Couser, N. L.; Pandya, A., Oculodentodigital Dysplasia: A Case Report and Major Review of the Eye and Ocular Adnexa Features of 295 Reported Cases. *Case reports in ophthalmological medicine* **2020,** 2020, 6535974.
13. Dwarakanathan, A.; Bhat, M.; Gn, S.; Shetty, S., Missense and deletion mutations in GJA1 causing oculodentodigital dysplasia in two Indian families. *Clin. Dysmorphol.* **2015,** 24, (4), 159-62.
14. Furuta, N.; Ikeda, M.; Hirayanagi, K.; Fujita, Y.; Amanuma, M.; Okamoto, K., A Novel GJA1 Mutation in Oculodentodigital Dysplasia with Progressive Spastic Paraplegia and Sensory Deficits. *Intern. Med.* **2012,** 51, (1), 93-98.
15. Pace, N. P.; Benoit, V.; Agius, D.; Grima, M. A.; Parascandalo, R.; Hilbert, P.; Borg, I., Two novel GJA1 variants in oculodentodigital dysplasia. *Molecular genetics & genomic medicine* **2019,** 7, (9), e882.
16. Richardson, R.; Donnai, D.; Meire, F.; Dixon, M. J., Expression of Gja1 correlates with the phenotype observed in oculodentodigital syndrome/type III syndactyly. *J. Med. Genet.* **2004,** 41, (1), 60.
17. Wang, Z.; Sun, L.; Wang, P.; Chen, C.; Zhang, A.; Wang, W.; Ding, X., Novel ocular findings in oculodentodigital dysplasia (ODDD): a case report and literature review. *Ophthalmic Genet.* **2019,** 40, (1), 54-59.
18. Rudenskaya, G. E.; Dyomina, N. A.; Bliznetz, E. A.; Khlebnikova, O. V.; Dadaly, E. L.; Polyakov, A. V., [Neurological presentations of oculodentodigital dysplasia]. *Zh. Nevrol. Psikhiatr. Im. S. S. Korsakova* **2018,** 118, (5), 85-91.
19. Richardson, R. J.; Joss, S.; Tomkin, S.; Ahmed, M.; Sheridan, E.; Dixon, M. J., A nonsense mutation in the first transmembrane domain of connexin 43 underlies autosomal recessive oculodentodigital syndrome. *J. Med. Genet.* **2006,** 43, (7), e37.
20. Debeer, P.; Van Esch, H.; Huysmans, C.; Pijkels, E.; De Smet, L.; Van de Ven, W.; Devriendt, K.; Fryns, J. P., Novel GJA1 mutations in patients with oculo-dento-digital dysplasia (ODDD). *Eur J Med Genet* **2005,** 48, (4), 377-87.

21. Park, D. Y.; Cho, S. Y.; Jin, D. K.; Kee, C., Clinical Characteristics of Autosomal Dominant GJA1 Missense Mutation Linked to Oculodentodigital Dysplasia in a Korean Family. *J. Glaucoma* **2019,** 28, (4), 357-362.
22. Kellermayer, R.; Keller, M.; Ratajczak, P.; Richardson, E.; Harangi, F.; Merei, E.; Melegh, B.; Kosztolanyi, G.; Richard, G., Bigenic connexin mutations in a patient with hidrotic ectodermal dysplasia. *Eur. J. Dermatol.* **2005,** 15, (2), 75-9.
23. Gabriel, L. A.; Sachdeva, R.; Marcotty, A.; Rockwood, E. J.; Traboulsi, E. I., Oculodentodigital dysplasia: new ocular findings and a novel connexin 43 mutation. *Arch. Ophthalmol.* **2011,** 129, (6), 781-4.
24. Tumminelli, G.; Di Donato, I.; Guida, V.; Rufa, A.; De Luca, A.; Federico, A., Oculodentodigital dysplasia with massive brain calcification and a new mutation of GJA1 gene. *Journal of Alzheimer's disease : JAD* **2016,** 49, (1), 27-30.
25. Jamsheer, A.; Sowinska-Seidler, A.; Socha, M.; Stembalska, A.; Kiraly-Borri, C.; Latos-Bielenska, A., Three novel GJA1 missense substitutions resulting in oculo-dento-digital dysplasia (ODDD) - further extension of the mutational spectrum. *Gene* **2014,** 539, (1), 157-61.
26. Itro, A.; Marra, A.; Urciuolo, V.; Difalco, P.; Amodio, A., Oculodentodigital dysplasia. A case report. *Minerva Stomatol.* **2005,** 54, (7-8), 453-9.
27. Izumi, K.; Lippa, A. M.; Wilkens, A.; Feret, H. A.; McDonald-McGinn, D. M.; Zackai, E. H., Congenital heart defects in oculodentodigital dysplasia: Report of two cases. *Am J Med Genet A* **2013,** 161a, (12), 3150-4.
28. Cavusoglu, D.; Dundar, N. O.; Arican, P.; Ozyilmaz, B.; Gencpinar, P., A hypomyelinating leukodystrophy with calcification: oculodentodigital dysplasia. *Acta Neurol. Belg.* **2020,** 120, (5), 1177-1179.
29. Vasconcellos, J. P.; Melo, M. B.; Schimiti, R. B.; Bressanim, N. C.; Costa, F. F.; Costa, V. P., A novel mutation in the GJA1 gene in a family with oculodentodigital dysplasia. *Arch. Ophthalmol.* **2005,** 123, (10), 1422-6.
30. Choi, J.; Yang, A.; Song, A.; Lim, M.; Kim, J.; Jang, J. H.; Park, K. T.; Cho, S.; Jin, D. K., Oculodentodigital Dysplasia with a Novel Mutation in GJA1 Diagnosed by Targeted Gene Panel Sequencing: A Case Report and Literature Review. *Ann. Clin. Lab. Sci.* **2018,** 48, (6), 776-781.
31. Pizzuti, A.; Flex, E.; Mingarelli, R.; Salpietro, C.; Zelante, L.; Dallapiccola, B., A homozygous GJA1 gene mutation causes a Hallermann-Streiff/ODDD spectrum phenotype. *Hum. Mutat.* **2004,** 23, (3), 286.
32. Honkaniemi, J.; Kalkkila, J.-P.; Koivisto, P.; Kähärä, V.; Latvala, T.; Simola, K., Letter to the editor: Novel GJA1 mutation in oculodentodigital dysplasia. *American Journal of Medical Genetics Part A* **2005,** 139A, (1), 48-49.
33. Kjaer, K. W.; Hansen, L.; Eiberg, H.; Leicht, P.; Opitz, J. M.; Tommerup, N., Novel Connexin 43 (GJA1) mutation causes oculo-dento-digital dysplasia with curly hair. *Am J Med Genet A* **2004,** 127A, (2), 152-7.
34. Wiest, T.; Herrmann, O.; Stogbauer, F.; Grasshoff, U.; Enders, H.; Koch, M. J.; Grond-Ginsbach, C.; Schwaninger, M., Clinical and genetic variability of oculodentodigital dysplasia. *Clin. Genet.* **2006,** 70, (1), 71-2.
35. Orosz, O.; Fodor, M.; Balogh, I.; Losonczy, G., Relative anterior microphthalmos in oculodentodigital dysplasia. *Indian J. Ophthalmol.* **2018,** 66, (2), 334-336.
36. Zeng, H.; Xie, L.; Tang, M.; Yang, Y.; Tan, Z., [A de novo GJA1 mutation identified by whole-exome sequencing in a patient with oculodentodigital dysplasia]. *Zhonghua Yi Xue Yi Chuan Xue Za Zhi* **2018,** 35, (2), 268-271.
37. Tasdelen, E.; Durmaz, C. D.; Karabulut, H. G., Autosomal Recessive Oculodentodigital Dysplasia: A Case Report and Review of the Literature. *Cytogenetic and genome research* **2018,** 154, (4), 181-186.
38. van Es, R. J.; Wittebol-Post, D.; Beemer, F. A., Oculodentodigital dysplasia with mandibular retrognathism and absence of syndactyly: a case report with a novel mutation in the connexin 43 gene. *Int. J. Oral Maxillofac. Surg.* **2007,** 36, (9), 858-60.
39. Vitiello, C.; D'Adamo, P.; Gentile, F.; Vingolo, E. M.; Gasparini, P.; Banfi, S., A novel GJA1 mutation causes oculodentodigital dysplasia without syndactyly. *Am J Med Genet A* **2005,** 133A, (1), 58-60.
40. Brice, G.; Ostergaard, P.; Jeffery, S.; Gordon, K.; Mortimer, P. S.; Mansour, S., A novel mutation in GJA1 causing oculodentodigital syndrome and primary lymphoedema in a three generation family. *Clin. Genet.* **2013,** 84, (4), 378-81.
41. Saint-Val, L.; Courtin, T.; Charles, P.; Verny, C.; Catala, M.; Schiffmann, R.; Boespflug-Tanguy, O.; Mochel, F., GJA1 Variants Cause Spastic Paraplegia Associated with Cerebral Hypomyelination. *AJNR. Am. J. Neuroradiol.* **2019,** 40, (5), 788-791.

42. Vreeburg, M.; de Zwart-Storm, E. A.; Schouten, M. I.; Nellen, R. G.; Marcus-Soekarman, D.; Devies, M.; van Geel, M.; van Steensel, M. A., Skin changes in oculo-dento-digital dysplasia are correlated with C-terminal truncations of connexin 43. *Am J Med Genet A* **2007,** 143, (4), 360-3.
43. Hu, Y.; Chen, I. P.; de Almeida, S.; Tiziani, V.; Do Amaral, C. M. R.; Gowrishankar, K.; Passos-Bueno, M. R.; Reichenberger, E. J., A Novel Autosomal Recessive GJA1 Missense Mutation Linked to Craniometaphyseal Dysplasia. *PLoS ONE* **2013,** 8, (8), e73576.
44. van Steensel, M. A.; Spruijt, L.; van der Burgt, I.; Bladergroen, R. S.; Vermeer, M.; Steijlen, P. M.; van Geel, M., A 2-bp deletion in the GJA1 gene is associated with oculo-dento-digital dysplasia with palmoplantar keratoderma. *Am J Med Genet A* **2005,** 132A, (2), 171-4.
45. Kumar, M.; Agarwal, T.; Kaur, P.; Khokhar, S.; Dada, R., Molecular and structural analysis of genetic variations in congenital cataract. *Mol. Vis.* **2013,** 19, 2436-50.
46. Yao, K.; Wang, W.; Zhu, Y.; Jin, C.; Shentu, X.; Jiang, J.; Zhang, Y.; Ni, S., A novel GJA3 mutation associated with congenital nuclear pulverulent and posterior polar cataract in a Chinese family. *Hum. Mutat.* **2011,** 32, (12), 1367-70.
47. Ma, A. S.; Grigg, J. R.; Ho, G.; Prokudin, I.; Farnsworth, E.; Holman, K.; Cheng, A.; Billson, F. A.; Martin, F.; Fraser, C.; Mowat, D.; Smith, J.; Christodoulou, J.; Flaherty, M.; Bennetts, B.; Jamieson, R. V., Sporadic and Familial Congenital Cataracts: Mutational Spectrum and New Diagnoses Using Next-Generation Sequencing. *Hum. Mutat.* **2016,** 37, (4), 371-84.
48. Addison, P. K.; Berry, V.; Holden, K. R.; Espinal, D.; Rivera, B.; Su, H.; Srivastava, A. K.; Bhattacharya, S. S., A novel mutation in the connexin 46 gene (GJA3) causes autosomal dominant zonular pulverulent cataract in a Hispanic family. *Mol. Vis.* **2006,** 12, 791-5.
49. Berry, V.; Ionides, A. C. W.; Pontikos, N.; Moghul, I.; Moore, A. T.; Cheetham, M. E.; Michaelides, M., Whole-genome sequencing reveals a recurrent missense mutation in the Connexin 46 (GJA3) gene causing autosomal-dominant lamellar cataract. *Eye (London, England)* **2018,** 32, 1661-1668.
50. Hansen, L.; Yao, W.; Eiberg, H.; Funding, M.; Riise, R.; Kjaer, K. W.; Hejtmancik, J. F.; Rosenberg, T., The congenital "ant-egg" cataract phenotype is caused by a missense mutation in connexin46. *Mol. Vis.* **2006,** 12, 1033-9.
51. Tong, J. J.; Sohn, B. C. H.; Lam, A.; Walters, D. E.; Vertel, B. M.; Ebihara, L., Properties of two cataract-associated mutations located in the NH(2) terminus of connexin 46. *Am J Physiol Cell Physiol* **2013,** 304, (9), C823-32.
52. Santhiya, S. T.; Kumar, G. S.; Sudhakar, P.; Gupta, N.; Klopp, N.; Illig, T.; Soker, T.; Groth, M.; Platzer, M.; Gopinath, P. M.; Graw, J., Molecular analysis of cataract families in India: new mutations in the CRYBB2 and GJA3 genes and rare polymorphisms. *Mol. Vis.* **2010,** 16, 1837-47.
53. Javadiyan, S.; Craig, J. E.; Souzeau, E.; Sharma, S.; Lower, K. M.; Mackey, D. A.; Staffieri, S. E.; Elder, J. E.; Taranath, D.; Straga, T.; Black, J.; Pater, J.; Casey, T.; Hewitt, A. W.; Burdon, K. P., High-Throughput Genetic Screening of 51 Pediatric Cataract Genes Identifies Causative Mutations in Inherited Pediatric Cataract in South Eastern Australia. *G3 (Bethesda)* **2017,** 7, (10), 3257-3268.
54. Ye, Y.; Wu, M.; Qiao, Y.; Xie, T.; Yu, Y.; Yao, K., Identification and preliminary functional analysis of two novel congenital cataract associated mutations of Cx46 and Cx50. *Ophthalmic Genet.* **2019,** 40, (5), 428-435.
55. Berry, V.; Ionides, A.; Pontikos, N.; Moghul, I.; Moore, A. T.; Quinlan, R. A.; Michaelides, M., Whole Exome Sequencing Reveals Novel and Recurrent Disease-Causing Variants in Lens Specific Gap Junctional Protein Encoding Genes Causing Congenital Cataract. *Genes* **2020,** 11, (5).
56. Devi, R. R.; Reena, C.; Vijayalakshmi, P., Novel mutations in GJA3 associated with autosomal dominant congenital cataract in the Indian population. *Mol. Vis.* **2005,** 11, 846-52.
57. Vidya, N. G.; Rajkumar, S.; Vasavada, A. R., Genetic investigation of ocular developmental genes in 52 patients with anophthalmia/microphthalmia. *Ophthalmic Genet.* **2018,** 39, (3), 344-352.
58. Jiang, H.; Jin, Y.; Bu, L.; Zhang, W.; Liu, J.; Cui, B.; Kong, X.; Hu, L., A novel mutation in GJA3 (connexin46) for autosomal dominant congenital nuclear pulverulent cataract. *Mol. Vis.* **2003,** 9, 579-83.
59. Guleria, K.; Sperling, K.; Singh, D.; Varon, R.; Singh, J. R.; Vanita, V., A novel mutation in the connexin 46 (GJA3) gene associated with autosomal dominant congenital cataract in an Indian family. *Mol. Vis.* **2007,** 13, 1657-65.
60. Yang, Z.; Li, Q.; Ma, X.; Zhu, S. Q., Mutation analysis in Chinese families with autosomal dominant hereditary cataracts. *Curr. Eye Res.* **2015,** 40, (12), 1225-31.

61. Zhou, Z.; Hu, S.; Wang, B.; Zhou, N.; Zhou, S.; Ma, X.; Qi, Y., Mutation analysis of congenital cataract in a Chinese family identified a novel missense mutation in the connexin 46 gene (GJA3). *Mol. Vis.* **2010,** 16, 713-9.
62. Bennett, T. M.; Shiels, A., A recurrent missense mutation in GJA3 associated with autosomal dominant cataract linked to chromosome 13q. *Mol. Vis.* **2011,** 17, 2255-62.
63. Zhang, X. H.; Da Wang, J.; Jia, H. Y.; Zhang, J. S.; Li, Y.; Xiong, Y.; Li, J.; Li, X. X.; Huang, Y.; Zhu, G. Y.; Rong, S. S.; Wormstone, M.; Wan, X. H., Mutation profiles of congenital cataract genes in 21 northern Chinese families. *Mol. Vis.* **2018,** 24, 471-477.
64. Ma, Z.; Zheng, J.; Yang, F.; Ji, J.; Li, X.; Tang, X.; Yuan, X.; Zhang, X.; Sun, H., Two novel mutations of connexin genes in Chinese families with autosomal dominant congenital nuclear cataract. *Br. J. Ophthalmol.* **2005,** 89, (11), 1535-7.
65. Guo, Y.; Yuan, L.; Yi, J.; Xiao, J.; Xu, H.; Lv, H.; Xiong, W.; Zheng, W.; Guan, L.; Zhang, J.; Xiang, H.; Qi, Y.; Deng, H., Identification of a GJA3 mutation in a Chinese family with congenital nuclear cataract using exome sequencing. *Indian J. Biochem. Biophys.* **2013,** 50, (4), 253-8.
66. Yang, G.; Xing, B.; Liu, G.; Lu, X.; Jia, X.; Wang, X.; Yu, H.; Fu, Y.; Zhao, J., A novel mutation in the GJA3 (connexin46) gene is associated with autosomal dominant congenital nuclear cataract in a Chinese family. *Mol. Vis.* **2011,** 17, 1070-3.
67. Li, B.; Liu, Y.; Liu, Y.; Guo, H.; Hu, Z.; Xia, K.; Jin, X., Identification of a GJA3 Mutation in a Large Family with Bilateral Congenital Cataract. *DNA Cell Biol.* **2016,** 35, (3), 135-9.
68. Gillespie, R. L.; O'Sullivan, J.; Ashworth, J.; Bhaskar, S.; Williams, S.; Biswas, S.; Kehdi, E.; Ramsden, S. C.; Clayton-Smith, J.; Black, G. C.; Lloyd, I. C., Personalized diagnosis and management of congenital cataract by next-generation sequencing. *Ophthalmology* **2014,** 121, (11), 2124-37.e1-2.
69. Hu, Y.; Gao, L.; Feng, Y.; Yang, T.; Huang, S.; Shao, Z.; Yuan, H., Identification of a novel mutation of the gene for gap junction protein α3 (GJA3) in a Chinese family with congenital cataract. *Mol. Biol. Rep.* **2014,** 41, (7), 4753-8.
70. Bennett, T. M.; Mackay, D. S.; Knopf, H. L.; Shiels, A., A novel missense mutation in the gene for gap-junction protein alpha3 (GJA3) associated with autosomal dominant "nuclear punctate" cataracts linked to chromosome 13q. *Mol. Vis.* **2004,** 10, 376-82.
71. Wang, L.; Chen, Y.; Chen, X.; Sun, X., Further evidence for P59L mutation in GJA3 associated with autosomal dominant congenital cataract. *Indian J. Ophthalmol.* **2016,** 64, (7), 508-12.
72. Hansen, L.; Mikkelsen, A.; Nurnberg, P.; Nurnberg, G.; Anjum, I.; Eiberg, H.; Rosenberg, T., Comprehensive mutational screening in a cohort of Danish families with hereditary congenital cataract. *Invest. Ophthalmol. Vis. Sci.* **2009,** 50, (7), 3291-303.
73. Sun, W.; Xiao, X.; Li, S.; Guo, X.; Zhang, Q., Mutation analysis of 12 genes in Chinese families with congenital cataracts. *Mol. Vis.* **2011,** 17, 2197-206.
74. Mackay, D.; Ionides, A.; Kibar, Z.; Rouleau, G.; Berry, V.; Moore, A.; Shiels, A.; Bhattacharya, S., Connexin46 mutations in autosomal dominant congenital cataract. *Am J Hum Genet* **1999,** 64, (5), 1357-64.
75. Burdon, K.; Wirth, M.; Mackey, D.; Russell-Eggitt, I.; Craig, J.; Elder, J.; Dickinson, J.; Sale, M., A novel mutation in the Connexin 46 gene causes autosomal dominant congenital cataract with incomplete penetrance. *J. Med. Genet.* **2004,** 41, (8), e106.
76. Guleria, K.; Vanita, V.; Singh, D.; Singh, J. R., A novel "pearl box" cataract associated with a mutation in the connexin 46 (GJA3) gene. *Mol. Vis.* **2007,** 13, 797-803.
77. Zhou, Z.; Wang, B.; Hu, S.; Zhang, C.; Ma, X.; Qi, Y., Genetic variations in GJA3, GJA8, LIM2, and age-related cataract in the Chinese population: a mutation screening study. *Mol. Vis.* **2011,** 17, 621-6.
78. Ren, Q.; Riquelme, M. A.; Xu, J.; Yan, X.; Nicholson, B. J.; Gu, S.; Jiang, J. X., Cataract-Causing Mutation of Human Connexin 46 Impairs Gap Junction, but Increases Hemichannel Function and Cell Death. *PLoS ONE* **2013,** 8, (9), e74732.
79. Zhang, L.; Qu, X.; Su, S.; Guan, L.; Liu, P., A novel mutation in GJA3 associated with congenital Coppock-like cataract in a large Chinese family. *Mol. Vis.* **2012,** 18, 2114-8.
80. Yuan, L.; Guo, Y.; Yi, J.; Xiao, J.; Yuan, J.; Xiong, W.; Xu, H.; Yang, Z.; Zhang, J.; Deng, H., Identification of a novel GJA3 mutation in congenital nuclear cataract. *Optom. Vis. Sci.* **2015,** 92, (3), 337-42.

81. Yao, Y.; Zheng, X.; Ge, X.; Xiu, Y.; Zhang, L.; Fang, W.; Zhao, J.; Gu, F.; Zhu, Y., Identification of a novel GJA3 mutation in a large Chinese family with congenital cataract using targeted exome sequencing. *PLoS ONE* **2017,** 12, (9).
82. Rees, M. I.; Watts, P.; Fenton, I.; Clarke, A.; Snell, R. G.; Owen, M. J.; Gray, J., Further evidence of autosomal dominant congenital zonular pulverulent cataracts linked to 13q11 (CZP3) and a novel mutation in connexin 46 (GJA3). *Hum. Genet.* **2000,** 106, (2), 206-9.
83. Ding, X.; Wang, B.; Luo, Y.; Hu, S.; Zhou, G.; Zhou, Z.; Wang, J.; Ma, X.; Qi, Y., A novel mutation in the connexin 46 (GJA3) gene associated with congenital cataract in a Chinese pedigree. *Mol. Vis.* **2011,** 17, 1343-9.
84. Li, Y.; Wang, J.; Dong, B.; Man, H., A novel connexin46 (GJA3) mutation in autosomal dominant congenital nuclear pulverulent cataract. *Mol. Vis.* **2004,** 10, 668-71.
85. Zhang, X.; Wang, L.; Wang, J.; Dong, B.; Li, Y., Coralliform cataract caused by a novel connexin46 (GJA3) mutation in a Chinese family. *Mol. Vis.* **2012,** 18, 203-10.
86. Musleh, M.; Hall, G.; Lloyd, I. C.; Gillespie, R. L.; Waller, S.; Douzgou, S.; Clayton-Smith, J.; Kehdi, E.; Black, G. C.; Ashworth, J., Diagnosing the cause of bilateral paediatric cataracts: comparison of standard testing with a next-generation sequencing approach. *Eye (London, England)* **2016,** 30, (9), 1175-81.
87. Ponnam, S. P. G.; Ramesha, K.; Matalia, J.; Tejwani, S.; Ramamurthy, B.; Kannabiran, C., Mutational screening of Indian families with hereditary congenital cataract. *Mol. Vis.* **2013,** 19, 1141-8.
88. Wang, K. J.; Zhu, S. Q., A novel p.F206I mutation in Cx46 associated with autosomal dominant congenital cataract. *Mol. Vis.* **2012,** 18, 968-73.
89. Micheal, S.; Niewold, I. T. G.; Siddiqui, S. N.; Zafar, S. N.; Khan, M. I.; Bergen, A. A. B., Delineation of Novel Autosomal Recessive Mutation in GJA3 and Autosomal Dominant Mutations in GJA8 in Pakistani Congenital Cataract Families. *Genes* **2018,** 9, (2).
90. Li, S.; Zhang, J.; Cao, Y.; You, Y.; Zhao, X., Novel mutations identified in Chinese families with autosomal dominant congenital cataracts by targeted next-generation sequencing. *BMC medical genetics* **2019,** 20, (1), 196.
91. Zhou, D.; Ji, H.; Wei, Z.; Guo, L.; Li, Y.; Wang, T.; Zhu, Y.; Dong, X.; Wang, Y.; He, L.; Xing, Q.; Zhang, L., A novel insertional mutation in the connexin 46 (gap junction alpha 3) gene associated with autosomal dominant congenital cataract in a Chinese family. *Mol. Vis.* **2013,** 19, 789-95.
92. Cui, X. K.; Zhu, K. K.; Zhou, Z.; Wan, S. M.; Dong, Y.; Wang, X. C.; Li, J.; Zhang, J.; Mu, H. M.; Qin, L.; Hu, Y. Z., A novel frameshift mutation in CX46 associated with hereditary dominant cataracts in a Chinese family. *International journal of ophthalmology* **2017,** 10, (5), 684-690.
93. Gollob, M. H.; Jones, D. L.; Krahn, A. D.; Danis, L.; Gong, X.-Q.; Shao, Q.; Liu, X.; Veinot, J. P.; Tang, A. S. L.; Stewart, A. F. R.; Tesson, F.; Klein, G. J.; Yee, R.; Skanes, A. C.; Guiraudon, G. M.; Ebihara, L.; Bai, D., Somatic Mutations in the Connexin 40 Gene (GJA5) in Atrial Fibrillation. *New England Journal of Medicine* **2006,** 354, (25), 2677-2688.
94. Yang, Y. Q.; Zhang, X. L.; Wang, X. H.; Tan, H. W.; Shi, H. F.; Jiang, W. F.; Fang, W. Y.; Liu, X., Connexin40 nonsense mutation in familial atrial fibrillation. *Int. J. Mol. Med.* **2010,** 26, (4), 605-10.
95. Sun, Y.; Yang, Y. Q.; Gong, X. Q.; Wang, X. H.; Li, R. G.; Tan, H. W.; Liu, X.; Fang, W. Y.; Bai, D., Novel germline GJA5/connexin40 mutations associated with lone atrial fibrillation impair gap junctional intercellular communication. *Hum Mutat* **2013,** 34, (4), 603-9.
96. Yang, Y. Q.; Liu, X.; Zhang, X. L.; Wang, X. H.; Tan, H. W.; Shi, H. F.; Jiang, W. F.; Fang, W. Y., Novel connexin40 missense mutations in patients with familial atrial fibrillation. *Europace* **2010,** 12, (10), 1421-7.
97. Lubkemeier, I.; Andrie, R.; Lickfett, L.; Bosen, F.; Stockigt, F.; Dobrowolski, R.; Draffehn, A. M.; Fregeac, J.; Schultze, J. L.; Bukauskas, F. F.; Schrickel, J. W.; Willecke, K., The Connexin40A96S mutation from a patient with atrial fibrillation causes decreased atrial conduction velocities and sustained episodes of induced atrial fibrillation in mice. *J. Mol. Cell. Cardiol.* **2013,** 65, 19-32.
98. Christophersen, I. E.; Holmegard, H. N.; Jabbari, J.; Sajadieh, A.; Haunso, S.; Tveit, A.; Svendsen, J. H.; Olesen, M. S., Rare variants in GJA5 are associated with early-onset lone atrial fibrillation. *The Canadian journal of cardiology* **2013,** 29, (1), 111-6.
99. Shi, H. F.; Yang, J. F.; Wang, Q.; Li, R. G.; Xu, Y. J.; Qu, X. K.; Fang, W. Y.; Liu, X.; Yang, Y. Q., Prevalence and spectrum of GJA5 mutations associated with lone atrial fibrillation. *Mol Med Rep* **2013,** 7, (3), 767-74.

100. Zhang, L.; Liang, Y.; Zhou, Y.; Zeng, H.; Jia, S.; Shi, J., A Missense Mutation in GJA8 Encoding Connexin 50 in a Chinese Pedigree with Autosomal Dominant Congenital Cataract. *Tohoku J. Exp. Med.* **2018,** 244, (2), 105-111.
101. Mackay, D. S.; Bennett, T. M.; Culican, S. M.; Shiels, A., Exome sequencing identifies novel and recurrent mutations in GJA8 and CRYGD associated with inherited cataract. *Hum Genomics* **2014,** 8, 19.
102. Javadiyan, S.; Lucas, S. E. M.; Wangmo, D.; Ngy, M.; Edussuriya, K.; Craig, J. E.; Rudkin, A.; Casson, R.; Selva, D.; Sharma, S.; Lower, K. M.; Meucke, J.; Burdon, K. P., Identification of novel mutations causing pediatric cataract in Bhutan, Cambodia, and Sri Lanka. *Molecular genetics & genomic medicine* **2018, 6**, (4), 555-64.
103. Astiazaran, M. C.; Garcia-Montano, L. A.; Sanchez-Moreno, F.; Matiz-Moreno, H.; Zenteno, J. C., Next generation sequencing-based molecular diagnosis in familial congenital cataract expands the mutational spectrum in known congenital cataract genes. *Am J Med Genet A* **2018,** 176, (12), 2637-2645.
104. Willoughby, C. E.; Arab, S.; Gandhi, R.; Zeinali, S.; Luk, D.; Billingsley, G.; Munier, F. L.; Heon, E., A novel GJA8 mutation in an Iranian family with progressive autosomal dominant congenital nuclear cataract. *J. Med. Genet.* **2003,** 40, (11), e124.
105. Wang, J.; Wang, H.; Wang, Y.; Chen, T.; Wu, X.; Jiang, Y., Two novel gap junction protein alpha 12 gene mutations in two Chinese patients with Pelizaeus-Merzbacher-like disease. *Brain Dev* **2010,** 32, (3), 236-43.
106. Ren, M.; Yang, X. G.; Dang, X. J.; Xiao, J. A., Exome sequencing identifies a novel mutation in GJA8 associated with inherited cataract in a Chinese family. *Graefes Arch. Clin. Exp. Ophthalmol.* **2017,** 255, (1), 141-151.
107. Dang, F. T.; Yang, F. Y.; Yang, Y. Q.; Ge, X. L.; Chen, D.; Zhang, L.; Yu, X. P.; Gu, F.; Zhu, Y. H., A novel mutation of p.F32I in GJA8 in human dominant congenital cataracts. *International journal of ophthalmology* **2016,** 9, (11), 1561-1567.
108. Sun, W.; Xiao, X.; Li, S.; Guo, X.; Zhang, Q., Mutational screening of six genes in Chinese patients with congenital cataract and microcornea. *Mol. Vis.* **2011,** 17, 1508-13.
109. Ceroni, F.; Aguilera-Garcia, D.; Chassaing, N.; Bax, D. A.; Blanco-Kelly, F.; Ramos, P.; Tarilonte, M.; Villaverde, C.; da Silva, L. R. J.; Ballesta-Martinez, M. J.; Sanchez-Soler, M. J.; Holt, R. J.; Cooper-Charles, L.; Bruty, J.; Wallis, Y.; McMullan, D.; Hoffman, J.; Bunyan, D.; Stewart, A.; Stewart, H.; Lachlan, K.; Fryer, A.; McKay, V.; Roume, J.; Dureau, P.; Saggar, A.; Griffiths, M.; Calvas, P.; Ayuso, C.; Corton, M.; Ragge, N. K.; Study, D. D. D., New GJA8 variants and phenotypes highlight its critical role in a broad spectrum of eye anomalies. *Hum. Genet.* **2019,** 138, (8-9), 1027-1042.
110. Zhu, Y.; Yu, H.; Wang, W.; Gong, X.; Yao, K., Correction: A Novel GJA8 Mutation (p.V44A) Causing Autosomal Dominant Congenital Cataract. *PLoS One* **2015,** 10, (5), e0125949.
111. Zhu, Y.; Yu, H.; Wang, W.; Gong, X.; Yao, K., A novel GJA8 mutation (p.V44A) causing autosomal dominant congenital cataract. *PLoS One* **2014,** 9, (12), e115406.
112. Devi, R. R.; Vijayalakshmi, P., Novel mutations in GJA8 associated with autosomal dominant congenital cataract and microcornea. *Mol. Vis.* **2006,** 12, 190-5.
113. Mohebi, M.; Chenari, S.; Akbari, A.; Ghassemi, F.; Zarei-Ghanavati, M.; Fakhraie, G.; Babaie, N.; Heidari, M., Mutation analysis of connexin 50 gene among Iranian families with autosomal dominant cataracts. *Iran J Basic Med Sci* **2017,** 20, (3), 288-293.
114. Vanita, V.; Singh, J. R.; Singh, D.; Varon, R.; Sperling, K., A novel mutation in GJA8 associated with jellyfish-like cataract in a family of Indian origin. *Mol. Vis.* **2008,** 14, 323-6.
115. Zhang, H.; Chen, Z.; He, K.; Chang, P.; Zhao, Y.; Huang, X.; Li, J.; Jin, Z.; Zhao, Y. E., Unique presentation of congenital cataract concurrent with microcornea, microphthalmia plus posterior capsule defect in monozygotic twins caused by a novel GJA8 mutation. *Eye (London, England)* **2019,** 33, (4), 686-689.
116. Minogue, P. J.; Tong, J. J.; Arora, A.; Russell-Eggitt, I.; Hunt, D. M.; Moore, A. T.; Ebihara, L.; Beyer, E. C.; Berthoud, V. M., A mutant connexin50 with enhanced hemichannel function leads to cell death. *Invest. Ophthalmol. Vis. Sci.* **2009,** 50, (12), 5837-45.
117. Arora, A.; Minogue, P. J.; Liu, X.; Addison, P. K.; Russel-Eggitt, I.; Webster, A. R.; Hunt, D. M.; Ebihara, L.; Beyer, E. C.; Berthoud, V. M.; Moore, A. T., A novel connexin50 mutation associated with congenital nuclear pulverulent cataracts. *J. Med. Genet.* **2008,** 45, (3), 155-60.
118. He, W.; Li, X.; Chen, J.; Xu, L.; Zhang, F.; Dai, Q.; Cui, H.; Wang, D. M.; Yu, J.; Hu, S.; Lu, S., Genetic linkage analyses and Cx50 mutation detection in a large multiplex Chinese family with hereditary nuclear cataract. *Ophthalmic Genet.* **2011,** 32, (1), 48-53.

119. Gunda, P.; Manne, M.; Adeel, S. S.; Kondareddy, R. K. R.; Tirunilai, P., Detection of c.139G>A (D47N) mutation in GJA8 gene in an extended family with inheritance of autosomal dominant zonular cataract without pulverulent opacities by exome sequencing. *J Genet* **2018,** 97, (4), 879-885.
120. Li, J.; Wang, Q.; Fu, Q.; Zhu, Y.; Zhai, Y.; Yu, Y.; Zhang, K.; Yao, K., A novel connexin 50 gene (gap junction protein, alpha 8) mutation associated with congenital nuclear and zonular pulverulent cataract. *Mol. Vis.* **2013,** 19, 767-74.
121. Yan, N.; Xiao, L.; Hou, C.; Guo, B.; Fan, W.; Deng, Y.; Ma, K., X-linked inheritances recessive of congenital nystagmus and autosomal dominant inheritances of congenital cataracts coexist in a Chinese family: a case report and literature review. *BMC medical genetics* **2019,** 20, (1), 41.
122. Lin, Y.; Liu, N. N.; Lei, C. T.; Fan, Y. C.; Liu, X. Q.; Yang, Y.; Wang, J. F.; Liu, B.; Yang, Z. L., [A novel GJA8 mutation in a Chinese family with autosomal dominant congenital cataract]. *Zhonghua Yi Xue Yi Chuan Xue Za Zhi* **2008,** 25, (1), 59-62.
123. Berry, V.; Mackay, D.; Khaliq, S.; Francis, P. J.; Hameed, A.; Anwar, K.; Mehdi, S. Q.; Newbold, R. J.; Ionides, A.; Shiels, A.; Moore, T.; Bhattacharya, S. S., Connexin 50 mutation in a family with congenital "zonular nuclear" pulverulent cataract of Pakistani origin. *Hum. Genet.* **1999,** 105, (1-2), 168-70.
124. Hadrami, M.; Bonnet, C.; Veten, F.; Zeitz, C.; Condroyer, C.; Wang, P.; Biya, M.; Sidi Ahmed, M. A.; Zhang, Q.; Cheikh, S.; Audo, I.; Petit, C.; Houmeida, A., A novel missense mutation of GJA8 causes congenital cataract in a large Mauritanian family. *Eur. J. Ophthalmol.* **2019,** 29, (6), 621-628.
125. Yu, Y.; Wu, M.; Chen, X.; Zhu, Y.; Gong, X.; Yao, K., Identification and functional analysis of two novel connexin 50 mutations associated with autosome dominant congenital cataracts. *Sci Rep* **2016,** 6, 26551.
126. Reis, L. M.; Tyler, R. C.; Muheisen, S.; Raggio, V.; Salviati, L.; Han, D. P.; Costakos, D.; Yonath, H.; Hall, S.; Power, P.; Semina, E. V., Whole exome sequencing in dominant cataract identifies a new causative factor, CRYBA2, and a variety of novel alleles in known genes. *Hum. Genet.* **2013,** 132, (7), 761-70.
127. Li, L.; Fan, D. B.; Zhao, Y. T.; Li, Y.; Yang, Z. B.; Zheng, G. Y., GJA8 missense mutation disrupts hemichannels and induces cell apoptosis in human lens epithelial cells. *Sci Rep* **2019,** 9, (1), 19157.
128. Wang, K. J.; Da Wang, J.; Chen, D. D.; Wang, M. Y.; Yun, B.; Zhu, S. Q., Characterization of a p.R76H mutation in Cx50 identified in a Chinese family with congenital nuclear cataract. *J. Formos. Med. Assoc.* **2020,** 119, (1 Pt 1), 144-149.
129. Vanita, V.; Hennies, H. C.; Singh, D.; Nurnberg, P.; Sperling, K.; Singh, J. R., A novel mutation in GJA8 associated with autosomal dominant congenital cataract in a family of Indian origin. *Mol. Vis.* **2006,** 12, 1217-22.
130. Arora, A.; Minogue, P. J.; Liu, X.; Reddy, M. A.; Ainsworth, J. R.; Bhattacharya, S. S.; Webster, A. R.; Hunt, D. M.; Ebihara, L.; Moore, A. T.; Beyer, E. C.; Berthoud, V. M., A novel GJA8 mutation is associated with autosomal dominant lamellar pulverulent cataract: further evidence for gap junction dysfunction in human cataract. *J. Med. Genet.* **2006,** 43, (1), e2.
131. Shiels, A.; Mackay, D.; Ionides, A.; Berry, V.; Moore, A.; Bhattacharya, S., A missense mutation in the human connexin50 gene (GJA8) underlies autosomal dominant "zonular pulverulent" cataract, on chromosome 1q. *Am J Hum Genet* **1998,** 62, (3), 526-32.
132. Ge, X. L.; Zhang, Y.; Wu, Y.; Lv, J.; Zhang, W.; Jin, Z. B.; Qu, J.; Gu, F., Identification of a novel GJA8 (Cx50) point mutation causes human dominant congenital cataracts. *Sci Rep* **2014,** 4, 4121.
133. Cui, X.; Zhou, Z.; Zhu, K.; Feng, R.; Han, J.; Li, M.; Wang, S.; Li, J.; Zhang, J.; Jiang, Q.; Zhang, W.; Mu, H.; Liu, Y.; Hu, Y., A Novel Cx50 Insert Mutation from a Chinese Congenital Cataract Family Impairs Its Cellular Membrane Localization and Function. *DNA Cell Biol.* **2018,** 37, (5), 449-456.
134. Min, H. Y.; Qiao, P. P.; Yan, Z. H.; Jiang, H. F.; Zhu, Y. P.; Du, H. Q.; Li, Q.; Wang, J. W.; Zhang, J.; Sun, J.; Yi, X.; Yang, L., Targeted Genes Sequencing Identified a Novel 15 bp Deletion on GJA8 in a Chinese Family with Autosomal Dominant Congenital Cataracts. *Chin. Med. J. (Engl).* **2016,** 129, (7), 860-7.
135. Patel, N.; Anand, D.; Monies, D.; Maddirevula, S.; Khan, A. O.; Algoufi, T.; Alowain, M.; Faqeih, E.; Alshammari, M.; Qudair, A.; Alsharif, H.; Aljubran, F.; Alsaif, H. S.; Ibrahim, N.; Abdulwahab, F. M.; Hashem, M.; Alsedairy, H.; Aldahmesh, M. A.; Lachke, S. A.; Alkuraya, F. S., Novel phenotypes and loci identified through clinical genomics approaches to pediatric cataract. *Hum. Genet.* **2017,** 136, (2), 205-225.

136. Hansen, L.; Yao, W.; Eiberg, H.; Kjaer, K. W.; Baggesen, K.; Hejtmancik, J. F.; Rosenberg, T., Genetic heterogeneity in microcornea-cataract: five novel mutations in CRYAA, CRYGD, and GJA8. *Invest. Ophthalmol. Vis. Sci.* **2007,** 48, (9), 3937-44.
137. Santana, A.; Waiswo, M., The genetic and molecular basis of congenital cataract. *Arq. Bras. Oftalmol.* **2011,** 74, (2), 136-42.
138. Prokudin, I.; Simons, C.; Grigg, J. R.; Storen, R.; Kumar, V.; Phua, Z. Y.; Smith, J.; Flaherty, M.; Davila, S.; Jamieson, R. V., Exome sequencing in developmental eye disease leads to identification of causal variants in GJA8, CRYGC, PAX6 and CYP1B1. *Eur. J. Hum. Genet.* **2014,** 22, (7), 907-15.
139. Hu, S.; Wang, B.; Zhou, Z.; Zhou, G.; Wang, J.; Ma, X.; Qi, Y., A novel mutation in GJA8 causing congenital cataract-microcornea syndrome in a Chinese pedigree. *Mol. Vis.* **2010,** 16, 1585-92.
140. Su, D.; Yang, Z.; Li, Q.; Guan, L.; Zhang, H.; E, D.; Zhang, L.; Zhu, S.; Ma, X., Identification and functional analysis of GJA8 mutation in a Chinese family with autosomal dominant perinuclear cataracts. *PLoS One* **2013,** 8, (3), e59926.
141. Ponnam, S. P.; Ramesha, K.; Tejwani, S.; Ramamurthy, B.; Kannabiran, C., Mutation of the gap junction protein alpha 8 (GJA8) gene causes autosomal recessive cataract. *J. Med. Genet.* **2007,** 44, (7), e85.
142. Kuo, D. S.; Sokol, J. T.; Minogue, P. J.; Berthoud, V. M.; Slavotinek, A. M.; Beyer, E. C.; Gould, D. B., Characterization of a variant of gap junction protein alpha8 identified in a family with hereditary cataract. *PLoS One* **2017,** 12, (8), e0183438.
143. Polyakov, A. V.; Shagina, I. A.; Khlebnikova, O. V.; Evgrafov, O. V., Mutation in the connexin 50 gene (GJA8) in a Russian family with zonular pulverulent cataract. *Clin. Genet.* **2001,** 60, (6), 476-8.
144. Graw, J.; Schmidt, W.; Minogue, P. J.; Rodriguez, J.; Tong, J. J.; Klopp, N.; Illig, T.; Ebihara, L.; Berthoud, V. M.; Beyer, E. C., The GJA8 allele encoding CX50I247M is a rare polymorphism, not a cataract-causing mutation. *Mol. Vis.* **2009,** 15, 1881-5.
145. Schmidt, W.; Klopp, N.; Illig, T.; Graw, J., A novel GJA8 mutation causing a recessive triangular cataract. *Mol. Vis.* **2008,** 14, 851-6.
146. Gao, X.; Cheng, J.; Lu, C.; Li, X.; Li, F.; Liu, C.; Zhang, M.; Zhu, S.; Ma, X., A novel mutation in the connexin 50 gene (GJA8) associated with autosomal dominant congenital nuclear cataract in a Chinese family. *Curr. Eye Res.* **2010,** 35, (7), 597-604.
147. Yan, M.; Xiong, C.; Ye, S. Q.; Chen, Y.; Ke, M.; Zheng, F.; Zhou, X., A novel connexin 50 (GJA8) mutation in a Chinese family with a dominant congenital pulverulent nuclear cataract. *Mol. Vis.* **2008,** 14, 418-24.
148. Chen, C.; Sun, Q.; Gu, M.; Liu, K.; Sun, Y.; Xu, X., A novel Cx50 (GJA8) p.H277Y mutation associated with autosomal dominant congenital cataract identified with targeted next-generation sequencing. *Graefes Arch. Clin. Exp. Ophthalmol.* **2015,** 253, (6), 915-24.
149. Kumar, M.; Agarwal, T.; Khokhar, S.; Kaur, P.; Roy, T. S.; Dada, R., Mutation screening and genotype phenotype correlation of alpha-crystallin, gamma-crystallin and GJA8 gene in congenital cataract. *Mol. Vis.* **2011,** 17, 693-707.
150. Senthil Kumar, G.; Dinesh Kumar, K.; Minogue, P. J.; Berthoud, V. M.; Kannan, R.; Beyer, E. C.; Santhiya, S. T., The E368Q Mutant Allele of GJA8 is Associated with Congenital Cataracts with Intrafamilial Variation in a South Indian Family. *Open access journal of ophthalmology* **2016,** 1, (1).
